# UNC93B1 promotes pancreatic cancer progression through modulation of cGAS–STING signaling

**DOI:** 10.3389/fimmu.2026.1718849

**Published:** 2026-02-04

**Authors:** Hao Yang, Yukun Li, Jing Su, Haiyan Zhang, Wenxin Zhu, Kanger Shen, Wei Xu

**Affiliations:** 1 Department of Medicine, The University of Hong Kong-Shenzhen Hospital, Shenzhen, China; 2 Department of Medicine, HuiYa Hospital of The First Affiliated Hospital, Sun Yat - sen University, Huizhou, China; 3 Department of Gastroenterology, Xuzhou Central Hospital, Southeast University, Xuzhou, China; 4 Department of Gastroenterology, Kunshan Third People’s Hospital, Suzhou, China; 5 Department of Gastroenterology, Huzhou Central Hospital, Huzhou, China

**Keywords:** cGAS-STING pathway, immune evasion, pancreatic cancer, tumor microenvironment, UNC93B1

## Abstract

**Background:**

Pancreatic ductal adenocarcinoma (PDAC) remains among the most lethal solid tumors, largely due to its intricate and immunosuppressive tumor microenvironment (TME). While single-cell sequencing technologies have begun to unravel the cellular heterogeneity of PDAC, a comprehensive understanding of how genetic determinants influence and are influenced by the TME is still lacking. To bridge this knowledge gap, our study employs an integrated multi-omics approach, incorporating single-cell transcriptomics, genomics, and proteomics, complemented by computational biology and machine learning. We aimed to delineate the core molecular drivers of PDAC pathogenesis, with subsequent *in vitro* functional validation focusing on the role of UNC93B1 in malignant phenotypes. The ultimate goal of this research is to inform the development of precise therapeutic strategies to enhance patient survival and quality of life.

**Methods:**

We assembled a comprehensive multi-omics dataset, including single-cell RNA-seq data from 22 PDAC samples (GSE154778, GSE212966), bulk transcriptomic cohorts (GSE28735, GSE62452), survival data from the TCGA-PAAD project (n=172), spatial transcriptomics, and genome-wide association study data (bbj-a-140, n=196,187). The single-cell data were processed using Seurat v5, which involved rigorous quality control, batch effect correction with Harmony, unsupervised clustering, and cell type annotation to characterize TME heterogeneity. Genetic susceptibility was mapped onto single-cell data using scPagwas to calculate trait-regulated scores (TRS) and identify trait-associated genes. Co-expression networks were constructed via high-diversity WGCNA (hdWGCNA), and key candidate genes were refined through survival analysis and a machine learning framework integrating LASSO regression, Random Forest, and Support Vector Machine algorithms. The functional role of the pivotal gene, UNC93B1, was systematically investigated through Gene Set Variation Analysis (GSVA), pseudotime trajectory inference (Monocle2), and cell-cell communication analysis (CellChat). *In vitro* validation was performed using four PDAC cell lines (PANC-1, BxPC-3, Capan-1, SW1990). Following qPCR confirmation of high UNC93B1 expression, a stable knockdown model (sh-UNC93B1) was generated in Capan-1 cells. Functional consequences were assessed using CCK-8, wound healing, transwell and colony formation assays. A subcutaneous xenograft model was established to evaluate tumor growth *in vivo*. Mechanistic insights were gained through flow cytometry for cell cycle analysis and molecular profiling of the cGAS-STING pathway, senescence markers (e.g., p16^INK4a), and epithelial-mesenchymal transition (EMT)-related genes.

**Results:**

Single-cell transcriptomic profiling delineated nine distinct cell populations within the PDAC TME. hdWGCNA identified three gene modules (8, 11, 16) positively associated with tumorigenesis. The intersection of these modules with differentially expressed genes yielded 320 candidates, which were subsequently filtered to 61 genes significantly linked to patient prognosis (P < 0.05) via Cox regression. Cross-validation across machine learning models and scPagwas analysis converged on UNC93B1 as the sole overlapping gene with consistent diagnostic and prognostic relevance. UNC93B1 was robustly upregulated in tumor tissues across independent datasets (TCGAxGTEx, bulk RNA-seq), a finding corroborated at the protein level by HPA and CPTAC data (P < 0.01). Its expression positively correlated with higher pathological grade and was spatially enriched within tumor regions. Functional enrichment analysis (GSVA) suggested that UNC93B1 is involved in the suppression of the cGAS-STING signaling axis. Pseudotime analysis indicated that UNC93B1 expression escalates along tumor progression trajectories. CellChat suggested strengthened intercellular communication networks in UNC93B1-high cells, particularly modulated by the cGAS-STING pathway. *In vitro*, UNC93B1 knockdown in Capan-1 cells significantly attenuated proliferative capacity (22.3% reduction in OD450 at 72h, P < 0.05), migratory ability (29.6% reduction in wound closure, P < 0.05), and clonogenic survival (342 fewer colonies, P < 0.01). Mechanistically, sh-UNC93B1 cells exhibited G1/S phase arrest (8.9% increase, P < 0.05), activation of the STING/IFN-β/CXCL10 cascade, elevated p16^INK4a expression, and a reversal of EMT, evidenced by downregulation of VIM and upregulation of CDH1. Consistently, *in vivo* xenograft experiments demonstrated that UNC93B1 silencing markedly impeded tumor growth, concomitant with reduced UNC93B1 protein and enhanced STING pathway activation. Critically, the tumor-suppressive phenotypes induced by UNC93B1 knockdown, including the inhibition of proliferation, migration, and clonogenicity, were largely reversed upon treatment with the selective STING inhibitor H-151, confirming that the observed functional consequences are causally mediated through the activation of the cGAS-STING pathway.

**Conclusion:**

By integrating multi-omics data, including GWAS and spatial transcriptomics, this study systematically defines a pivotal role for UNC93B1 in PDAC progression. Our findings demonstrate that UNC93B1 is associated with an immunosuppressive TME and facilitates metastatic spread, potentially through inhibiting the cGAS-STING-mediated innate immunity pathway. The strong correlation between UNC93B1 overexpression and adverse clinical outcomes underscores its potential as a dual diagnostic biomarker and therapeutic target. This work not only provides a mechanistic foundation for novel precision immunotherapies in PDAC but also establishes a robust methodological paradigm for multi-omics-driven discovery in oncology.

## 1 Introduction

Pancreatic ductal adenocarcinoma (PDAC) is among the most lethal malignancies of the digestive system, accounting for the third-highest number of cancer-related deaths in developed nations and demonstrating a persistently low five-year survival rate under 10% (1). The global burden of PDAC is escalating, with current projections indicating it will become the second leading cause of cancer mortality by 2030 (2). This dismal prognosis is attributable to a constellation of aggressive biological traits, including early asymptomatic progression, a profoundly fibrotic and immunosuppressive tumor microenvironment (TME), significant heterogeneity, and potent immune evasion mechanisms (3, 4). These factors collectively contribute to the profound resistance of PDAC to conventional therapies, as well as to emerging immunotherapies such as PD-1/PD-L1 checkpoint inhibitors (5, 6). Consequently, a deeper understanding of the molecular drivers of PDAC, particularly the dynamic crosstalk between neoplastic cells and the TME, is critical for developing effective therapeutic strategies.

The PDAC TME is a complex ecosystem dominated by a dense extracellular matrix (ECM), infiltrating immune cells like tumor-associated macrophages (TAMs) and myeloid-derived suppressor cells (MDSCs), and activated cancer-associated fibroblasts (CAFs) (7–9). This stroma creates both a physical barrier, through high mechanical pressure and hypoxia that limits drug delivery, and a molecular barrier, via immunosuppressive cytokines including TGF-β and IL-10 (10) (11). CAFs further remodel the ECM into a desmoplastic scaffold through the secretion of fibronectin and collagens (12–14), while simultaneously promoting metastasis through CXCL12/CXCR4-mediated activation of the PI3K-AKT pathway. Meanwhile, TAMs contribute to immune suppression by depleting local L-arginine via arginase-1 (ARG1) and expressing PD-L1 to inhibit CD8+ T-cell function (15). Despite these insights, the central regulatory nodes and the precise molecular dialogue orchestrating this network are not fully elucidated, presenting a major obstacle to personalized medicine.

The advent of single-cell RNA sequencing (scRNA-seq) has provided unprecedented resolution for deconstructing tumor heterogeneity (16). In cancers such as breast cancer and melanoma, this technology has uncovered rare cell subpopulations—for instance, chemotherapy-resistant dormant cells driven by Wnt signaling—that are linked to therapeutic failure (17, 18). Applying scRNA-seq to PDAC, however, presents distinct challenges: (1) the extensive fibrosis impedes high-quality cell dissociation, often leading to under-representation of epithelial cells and the potential loss of critical malignant clones; (2) many studies remain focused on individual cell compartments (e.g., T cells or CAFs), failing to capture the systematic cooperativity across the entire TME; and (3) the functional connection between genetic susceptibility loci identified through genome-wide association studies (GWAS) and cellular phenotypes at single-cell resolution remains largely unmapped, hindering the translation of genetic findings into pathological mechanisms.

Multi-omics integration has emerged as a powerful paradigm for deciphering the core regulatory circuits of oncogenesis. Pioneering work by Collison (19), Moffitt (20), and Bailey (21) has established foundational molecular subtypes of PDAC (e.g., “basal-like,” “classical”), paving the way for stratified treatment approaches. Nevertheless, bulk sequencing averages out cell-type-specific signals, while the inherent sparsity of scRNA-seq data complicates its integration with population-level genetics. Novel computational frameworks, such as scPagwas and high-diversity weighted gene co-expression network analysis (hdWGCNA), are designed to bridge this gap. scPagwas, for instance, has successfully mapped Alzheimer’s disease genetic risk to microglial subpopulations (22). Yet, the application of these advanced integrative methods in PDAC is still in its infancy, demanding rigorous biological validation to realize their clinical potential.

UNC93B1, an endoplasmic reticulum-resident transmembrane protein, is primarily characterized as a critical regulator of endosomal Toll-like receptor (TLR3, TLR7, TLR9) trafficking, thereby playing a fundamental role in innate immune sensing of nucleic acids. A growing body of evidence suggests its involvement in oncogenesis. UNC93B1 overexpression is associated with adverse outcomes in acute myeloid leukemia (23) and has been shown to drive oral squamous cell carcinoma progression through the upregulation of granulocyte colony-stimulating factor (G-CSF) (24). Notably, a recent seminal study identified UNC93B1 as a binding partner of STING, promoting its lysosomal degradation and thereby acting as a potent negative regulator of the cGAS-STING innate immune pathway—a mechanism implicated in tumor immune evasion (25). Despite these advances, the expression profile, functional significance, and TME-modulatory role of UNC93B1 in PDAC remain entirely unknown. Furthermore, its potential influence on epigenetic or metabolic reprogramming during cancer progression warrants systematic investigation.

In this study, we implement a comprehensive strategy that synergizes multi-omics discovery with experimental validation to define the role of UNC93B1 in PDAC. By integrating single-cell transcriptomics, GWAS, and proteomic data, we identified UNC93B1 as a pivotal risk gene strongly linked to patient prognosis. We subsequently employed a suite of *in vitro* and *in vivo* functional assays to validate its oncogenic properties and delineate the mechanistic basis of UNC93B1 in driving PDAC progression.

## 2 Methods

### 2.1 Data acquisition and preprocessing

Single-cell RNA sequencing (scRNA-seq) data from pancreatic cancer specimens were sourced from the Gene Expression Omnibus (GEO) under accessions GSE154778 and GSE212966. The GSE154778 cohort included 16 samples (10 primary tumors, 6 metastatic lesions) profiled on the Illumina NovaSeq 4000 platform. The GSE212966 dataset contained 12 samples (6 tumor-adjacent tissue pairs) sequenced on the Illumina NovaSeq 6000. As comprehensive tumor subtype annotations were unavailable for GSE212966, this dataset was reserved for preliminary analyses and excluded from final integrative modeling. Expression matrices for both datasets were generated using the CellRanger pipeline (10x Genomics). Bulk transcriptomic profiles and corresponding clinical survival data for 172 PDAC samples were obtained from The Cancer Genome Atlas (TCGA-PAAD) after removing 14 samples with incomplete metadata. Genome-wide association study (GWAS) summary statistics (ID: bbj-a-140) were acquired from the IEU OpenGWAS project, comprising 196,187 individuals (442 cases, 195,745 controls) and 8,885,075 single nucleotide polymorphisms (SNPs) based on the GRCh37/hg19 assembly. Additional bulk transcriptomic datasets (GSE28735, GSE62452), generated on the GPL6244 platform and encompassing 220 samples (114 tumors, 106 adjacent tissues), were incorporated. For independent validation, the TCGAxGTEx cohort (183 tumors, 167 normal tissues) was retrieved from the UCSC Xena portal.

### 2.2 Single-cell data analysis

Raw gene-barcode matrices were processed using Seurat v5. Initial quality control retained genes detected in a minimum of three cells (min.cells=3) and cells expressing at least 200 genes (min.features=200). Cells were filtered based on the following thresholds: total UMI counts (nCount_RNA) below 1,000; unique gene counts (nFeature_RNA) outside the 200-7,000 range; mitochondrial gene ratio (percent.mt) exceeding 20%; ribosomal gene expression outliers (extreme 1%); or anomalously high UMI/feature counts (top 3%). Quality metrics were visualized via violin and feature scatter plots. Data normalization was performed using a global-scaling LogNormalize method (scale factor 10,000). The top 4,000 highly variable genes (HVGs) were selected using a variance-stabilizing transformation (vst). Principal component analysis (PCA) was conducted on HVGs, with significant principal components (PCs) identified through elbow plots and JackStraw resampling (100 replicates). Batch effects were corrected using Harmony, and non-linear dimensionality reduction (UMAP/t-SNE) was applied using the first 20 Harmony-corrected PCs. Cell clusters were identified via Leiden graph-based clustering (resolution=0.8) on a K-nearest neighbor (KNN) graph. Cell type annotation was performed by integrating automated predictions from SingleR (referenced against the HumanPrimaryCellAtlas) with manual curation using established marker genes (e.g., “EPCAM/KRT19”for epithelial cells; “CD3D/CD8A” for T cells). Cell type proportions across tissue types were visualized with stacked bar plots, and cluster-specific marker genes were identified using Wilcoxon rank-sum tests.

Spatial transcriptomic data were processed within the Seurat framework, applying SCTransform normalization, PCA, and UMAP for dimensionality reduction. Cell type assignments for spatial spots were inferred from author-provided annotations. Spatial expression patterns of key genes were visualized using the FeaturePlot function.

### 2.3 Integration of GWAS and single-cell data

The scPagwas algorithm (22, 26) was implemented to map GWAS-derived genetic associations onto the single-cell transcriptomic landscape. Essential fields (genomic coordinates, effect size, standard error) were extracted from GWAS VCF files, and missing beta coefficients were derived as β = ES/SE. Genomic coordinates were lifted over from hg19 to hg38 using the rtracklayer package. Duplicate and non-significant SNPs (P ≥ 0.05) were filtered out. Trait regulatory scores (TRS) for KEGG pathways were computed and projected onto single-cell clusters. Differential TRS across cell subpopulations were assessed using violin plots and Wilcoxon rank-sum tests.

### 2.4 Construction of gene co-expression networks

High-dimensional weighted gene co-expression network analysis (hdWGCNA) was employed to identify trait-associated gene modules within the PDAC microenvironment. A high-confidence expression matrix was subset from the processed Seurat object, retaining genes detected in ≥5% of cells. To address data sparsity, metacells (25 cells per group, maximum shared cells=10) were generated using the MetacellsByGroups function, stratified by cell type and tissue phenotype. Metacell expression values were log-normalized and Z-score transformed. Harmony was applied to correct for technical variance prior to network construction. A soft-thresholding power of 5 was selected to achieve a scale-free topology (R² > 0.8). A topological overlap matrix was built, and dynamic tree cutting delineated co-expression modules (minimum module size=30; grey module discarded). Module eigengenes (MEs) were harmonized to generate hybrid module eigengenes (hMEs). Differentially expressed genes (DEGs) between tumor and adjacent tissues were identified using the Wilcoxon test (P < 0.05). Key candidate genes were defined as the intersection of high kME (module membership) genes and the DEG list. Functional enrichment of modules for KEGG and Gene Ontology terms was performed.

### 2.5 Candidate gene screening and machine learning validation

A pool of 320 candidate genes was established from the intersection of hdWGCNA-derived trait-relevant genes and epithelial cell-enriched markers. Univariate Cox proportional hazards regression (P < 0.05) applied to TCGA survival data identified 61 prognosis-associated genes. These were subsequently refined through a tripartite machine learning framework: (1) LASSO regression (glmnet v4.1-8) with 10-fold cross-validation to select features minimizing the binomial deviance; (2) Random Forest (randomForest v4.7-1.2, 500 trees) to rank genes by mean decrease in Gini impurity; and (3) Support Vector Machine (SVM; e1071 package) with recursive feature elimination (10 iterations, retaining the top 100 features per round). The consensus of the top 20 genes from each algorithm defined the Machine Learning Diagnostic Set (MLDS). In parallel, the scPagwas-Associated Diagnostic Set (PADS) was generated by intersecting candidate genes with scPagwas-identified genes (|R| > 0.1, P < 0.05). Diagnostic performance was evaluated by receiver operating characteristic (ROC) analysis in bulk datasets (GSE28735, GSE62452) and the independent TCGAxGTEx cohort. The overlapping gene between MLDS and PADS, UNC93B1, was selected as the central candidate for subsequent functional investigation.

### 2.6 Multi-omics validation of UNC93B1

UNC93B1 expression was validated at the transcriptomic and proteomic levels using data from the Human Protein Atlas (HPA, v23.0) and GTEx (27). Normalized expression (nTPM) for each gene was defined as the maximum nTPM value across all profiled tissues and cancer types. For anatomical structures with sub-regions, the maximum value among sub-regions was assigned to the parent tissue. Results were visualized using lollipop plots. Immunohistochemical (IHC) staining images for UNC93B1 in normal and PDAC tissues were retrieved from HPA. Proteomic validation utilized the Clinical Proteomic Tumor Analysis Consortium (CPTAC) pan-cancer dataset (28). Samples were stratified into tumor and normal groups based on clinical annotations, and differential protein abundance was assessed using the Wilcoxon test. To interrogate the functional state associations of UNC93B1, single-cell functional spectra from the CancerSEA database (29)—which quantifies 14 distinct functional states (e.g., apoptosis, proliferation, invasion)—were analyzed. Pathway activity scores were computed using Gene Set Variation Analysis (GSVA) (30) with the single-sample GSEA (ssGSEA) algorithm. Z-score normalization (31) was applied, and Pearson correlation coefficients between UNC93B1 expression and functional state scores were calculated. Associations between UNC93B1 expression and tumor grade in the TCGA cohort, as well as its spatial expression pattern, were further examined.

### 2.7 Pseudotime trajectory inference

Developmental trajectories were reconstructed using Monocle2 (v2.36.0). Significantly differentially expressed genes (P < 0.01), identified using a negative binomial generalized linear model, served as ordering genes. Dimensionality reduction was performed with the DDRTree algorithm, and cells were ordered in pseudotime using the orderCells function. Resulting trajectories were visualized, colored by cell type, state, or pseudotime value. Expression dynamics of UNC93B1 and established PDAC-associated genes (CDK6, SMAD4) along the pseudotime axis were plotted using the plot_genes_in_pseudotime function to assess their correlation with tumor progression.

### 2.8 Functional enrichment analysis using GSVA

Pathway enrichment analysis was conducted with Gene Set Variation Analysis (GSVA) to infer biological pathway activity from transcriptomic data. The analysis was performed in R using the GSVA package (v1.50.0) with the single-sample GSEA (ssGSEA) algorithm on raw count matrices. The c2.cp.kegg.v7.4.symbols.gmt gene set collection from the Molecular Signatures Database was employed. Differential pathway activity between tumor and normal tissue groups was identified using linear models implemented in the limma package, with empirical Bayes moderation (eBayes function) to compute moderated t-statistics. Pathways were deemed significantly differentially active with an absolute t-value > 2 and an adjusted p-value (Padj) < 0.05. This approach enabled the identification of key molecular networks modulated by UNC93B1, with a specific focus on its functional impact within epithelial cells.

### 2.9 Inference of cell-cell communication

Cell-cell communication networks were systematically reconstructed using the CellChat package (v1.6.0), which leverages a curated database of ligand-receptor interactions. The analysis began by identifying overexpressed ligands and receptors within each cell group using the identifyOverExpressedGenes function (threshold: expressed in >25% of cells per group). Interaction probabilities were then computed with computeCommunProb (trimming value = 0.1 to weight the top 10% of interactions), and the network was filtered to retain only interactions involving a minimum of 10 cells. These interactions were aggregated at the signaling pathway level to discern dominant communication flows. This analysis was instrumental in identifying UNC93B1-associated alterations in intercellular crosstalk, particularly within the epithelial compartment, providing mechanistic multi-omics insights into metastatic processes.

### 2.10 Cell culture

Human pancreatic cancer cell lines (PANC-1, BxPC-3, Capan-1, SW1990) were procured from Wuhan Procell Life Science & Technology Co., Ltd. All cell lines were maintained in RPMI-1640 medium, supplemented with 10% (v/v) fetal bovine serum (FBS) and 1% (v/v) penicillin-streptomycin, and incubated at 37 °C in a humidified atmosphere of 5% CO_2_. Cells were routinely passaged using trypsin-EDTA upon reaching 80-90% confluence during the logarithmic growth phase.

### 2.11 Generation of stable knockdown cell lines

Stable UNC93B1 knockdown was achieved via lentiviral transduction. Lentiviral particles were packaged using a third-generation system comprising the pLKO.1-EGFP-Puro transfer plasmid (encoding shRNA, an EGFP reporter, and a puromycin resistance gene), psPAX2 (packaging plasmid), and pMD2.G (envelope plasmid), all sourced from Shanghai Sangon Biotech. Three distinct shRNA sequences targeting UNC93B1 were designed (see **Table 1** for sequences).

**Table 1:** shRNA sequences.

SequenceID	Strands	Sequence (5’-3’)
SIR954-1	Passenger	CCGGGCGAGGTGAAGTATGGCAACATCTCTTGAATGTTG CCATACTTCACCTCGCTTTTTTG
SIR954-1	Guide	AATTCAAAAAAGCGAGGTGAAGTATGGCAACATTCAAGA GATGTTGCCATACTTCACCTCGC
SIR954-2	Passenger	CCGGGCTTCTTCCATCTGAGCTTCGTCTCTTGAACGAAGC TCAGATGGAAGAAGCTTTTTTG
SIR954-2	Guide	AATTCAAAAAAGCTTCTTCCATCTGAGCTTCGTTCAAGAG ACGAAGCTCAGATGGAAGAAGC
SIR954-3	Passenger	CCGGGCCACCTCGTGCCTTTCTTTATCTCTTGAATAAAGAA AGGCACGAGGTGGCTTTTTTG
SIR954-3	Guide	AATTCAAAAAAGCCACCTCGTGCCTTTCTTTATTCAAGAGA TAAAGAAAGGCACGAGGTGGC

For transduction, target cells were seeded in 24-well plates at 30-50% confluence. After 24 hours, cells were transduced with lentivirus at a pre-optimized multiplicity of infection (MOI), determined from a gradient test that balanced high transduction efficiency (≥70% GFP-positive cells observed via fluorescence microscopy) with cell viability (>85% as assessed by CCK-8 assay). Following 48 hours of incubation, stable polyclonal populations were selected by treatment with 0.4 μg/mL puromycin for 14 days; this concentration was predetermined to eliminate >90% of wild-type cells. Stable clones were further isolated via limiting dilution. Knockdown efficiency was confirmed by quantitative PCR (qPCR), resulting in the establishment of sh-NC (non-targeting control) and sh-UNC93B1 stable cell lines.

### 2.12 Quantitative PCR analysis

Total RNA was extracted from cells using the RNAeasy Isolation Reagent (Vazyme, Nanjing) according to the manufacturer’s instructions. Briefly, cells were lysed directly in the culture dish, and the lysate was homogenized before the addition of nuclease-free water. After centrifugation, the aqueous phase was mixed with an equal volume of isopropanol to precipitate RNA. The RNA pellet was washed with 75% ethanol, air-dried, and resuspended in nuclease-free water. RNA purity and concentration were determined using a NanoDrop 2000 spectrophotometer, with all samples having an A260/A280 ratio between 1.8 and 2.0. Genomic DNA removal and cDNA synthesis were performed using the HiScript III RT SuperMix (Vazyme). qPCR was carried out using the ChamQ Universal SYBR qPCR Master Mix (Vazyme) in 10 μL reaction volumes on a real-time PCR system. Primer sequences for UNC93B1, genes involved in the cGAS-STING pathway (GAS, IFN-α, p16INK4a, STING), epithelial-mesenchymal transition (EMT) markers (VIM, CDH1), and the reference gene GAPDH are listed in **Table 2** (Shanghai Sangon Biotech). Relative gene expression was calculated using the 2^(-ΔΔCt) method.

**Table 2:** qPCR primer.

Genes	Sequences (5’-3’)	Notes
GAS	AGCAACTACGACTAAAGCCAT	Forward primer
	CTTCTTTGTTTTCACAGCACGTT	Reverse primer
IFN-α	AAGCAGCAATTTTCAGTGTCA	Forward primer
	CCTCCCATTCAATTGCCACA	Reverse primer
p16INK4a	GCCGACCCCGCCACTCTCACC	Forward primer
	TGCAGCACCACCAGCGTGTCC	Reverse primer
STING	GGAATTTCAACGTGGCCCAT	Forward primer
	TGGCTCACTGCACCCCGTA	Reverse primer
CDH1	GGTATCTTCCCCGCCCTG	Forward primer
	CTTCATAGTCAAACACGAGCAG	Reverse primer
VIM	AAATGGCTCGTCACCTTCGT	Forward primer
	AGGGCCATCTTAACATTGAGCA	Reverse primer
UNC93B1	TGCCTCGGGTCCTGCAACACA	Forward primer
	AGCACCGCCAGCTTAGCCTT	Reverse primer
GAPDH	CAGGAGGCATTGCTGATGAT	Forward primer
	GAAGGCTGGGGCTCATTT	Reverse primer

### 2.13 Functional phenotypic assays

Cell Proliferation: Cell viability and proliferation were assessed using the Cell Counting Kit-8 (CCK-8, HyClone). sh-NC and sh-UNC93B1 Capan-1 cells were seeded in 96-well plates at a density of 3,000 cells per well. At 24, 48, and 72-hour time points, 10 μL of CCK-8 reagent was added to each well, followed by a 2-hour incubation. The absorbance at 450 nm was measured using a microplate reader.

Cell Migration: A wound healing assay was used to evaluate migratory capacity. Confluent monolayers of cells in 6-well plates were scratched with a 200 μL sterile pipette tip. After washing with PBS to remove detached cells, fresh serum-free medium was added. Wound areas were imaged at 0 and 12 hours. The migration rate was quantified using ImageJ software by measuring the change in the wound area over time.

Cell Invasion: Cell invasive and migratory capacities were evaluated using Transwell chambers (8-μm pore size; Corning). For the invasion assay, chambers were pre-coated with Matrigel (BD Biosciences) at 37°C for 1 hour. Briefly, sh-NC and sh-UNC93B1 Capan-1 cells were harvested and resuspended in serum-free RPMI-1640 medium. A total of 5 * 10^4^ cells in 200 μL of serum-free medium were seeded into the upper chamber. The lower chamber was filled with 600 μL of complete medium containing 10% FBS as a chemoattractant. After incubation for 24 hours at 37°C in 5% CO_2_, non-invaded/migrated cells on the upper surface of the membrane were carefully removed with a cotton swab. Cells that had invaded/migrated to the lower surface were fixed with 4% paraformaldehyde for 15 minutes, stained with 0.1% crystal violet for 20 minutes, and then washed with PBS. Stained cells were imaged under a light microscope (Olympus), and the number of cells in five random fields per chamber was counted using ImageJ software (National Institutes of Health). Experiments were performed in triplicate.

Clonogenic Survival: For the colony formation assay, 3,000 cells per well were seeded into 6-well plates and cultured for 8 days, with the medium refreshed every 48 hours. The resulting colonies were fixed with 4% paraformaldehyde, stained with 0.1% crystal violet, and counted manually. The colony formation rate was calculated as (number of colonies/initial cell count) × 100.

### 2.14 Cell cycle analysis by flow cytometry

Cells were harvested during the logarithmic growth phase, fixed in ice-cold 75% ethanol overnight at 4 °C, and then washed with PBS. Fixed cells were resuspended in a staining solution containing propidium iodide (PI) and RNase A, and incubated for 30 minutes at 37 °C in the dark. Cell cycle distribution was analyzed using a flow cytometer, and the percentage of cells in G0/G1, S, and G2/M phases was determined using ModFit LT software.

### 2.15 *In vivo* xenograft model

All animal procedures were approved by the Institutional Animal Care and Use Committee (Approval No: XZXY-LK-20241127-0197). Female Balb/C nude mice (8 weeks old, 20 ± 2 g) were purchased from Zhuhai BST Biological Technology Co., Ltd. and housed under specific pathogen-free (SPF) conditions.

Mice were randomly divided into two groups (sh-NC and sh-UNC93B1). Capan-1 cells (2 × 10^6^) in 100 μL of PBS were injected subcutaneously into the right axilla of each mouse. Tumor growth was monitored by measuring the length (a) and width (b) with calipers every two days starting from day 5 post-inoculation. Tumor volume was calculated using the formula V = (a × b²)/2. Mouse body weight was recorded simultaneously. On day 15, all mice were euthanized, and the tumors were excised, weighed, and photographed. Tumor tissues were fixed in 4% paraformaldehyde for subsequent histological analysis.

### 2.16 Immunohistochemistry

Subcutaneous tumor tissues were fixed in 4% paraformaldehyde, dehydrated through a graded ethanol series, cleared in xylene, and embedded in paraffin. Sections (4 μm thick) were deparaffinized and rehydrated. Antigen retrieval was performed by heating the sections in sodium citrate buffer (pH 6.0) using a pressure cooker. Endogenous peroxidase activity was quenched with 3% H_2_O_2_. After blocking with normal serum, sections were incubated overnight at 4 °C with primary antibodies against CXCL10 (1:500), UNC93B1 (1:200), and STING (1:1000). Following washing, sections were incubated with an HRP-conjugated secondary antibody (1:1000) for 1 hour at room temperature. Signal was developed using a diaminobenzidine (DAB) substrate kit, and sections were counterstained with hematoxylin. Stained sections were dehydrated, cleared, and mounted for microscopic examination.

### 2.17 STING inhibition rescue assays

To establish a causal link between UNC93B1 knockdown-induced phenotypes and cGAS-STING pathway activation, rescue experiments were performed using the selective STING inhibitor H-151 (MedChemExpress). Capan-1 and PANC-1 cells were divided into six experimental groups: (1) Wild-type (WT), (2) UNC93B1-overexpressing (OE-UNC93B1), (3) stable knockdown with shRNA-1 (sh-1), (4) sh-1 treated with 2 μM H-151 (sh-1+H-151), (5) stable knockdown with shRNA-2 (sh-2), and (6) sh-2 treated with 2 μM H-151 (sh-2+H-151). For groups receiving H-151, the inhibitor was added to the culture medium 24 hours post-seeding and maintained throughout the assay duration. Transwell migration, colony formation, and CCK-8 proliferation assays were then conducted as described in sections 2.13 and 2.14.

### 2.18 Statistical analysis

Statistical analyses were performed using R software (v4.3.2) and GraphPad Prism (v10.0). For bioinformatics analyses, standard pipelines for differential expression, co-expression networks, and machine learning were applied. Data from experimental replicates are presented as mean ± standard deviation (SD). Comparisons between two groups were analyzed using an unpaired two-tailed Student’s t-test. Multiple group comparisons were conducted using one-way or two-way ANOVA, as appropriate. For non-parametric data from IHC scoring, the Mann-Whitney U test was used. A p-value of less than 0.05 was considered statistically significant.

## 3 Results

### 3.1 A Single-cell transcriptomic atlas reveals the cellular architecture of PDAC

**Figure 1:**
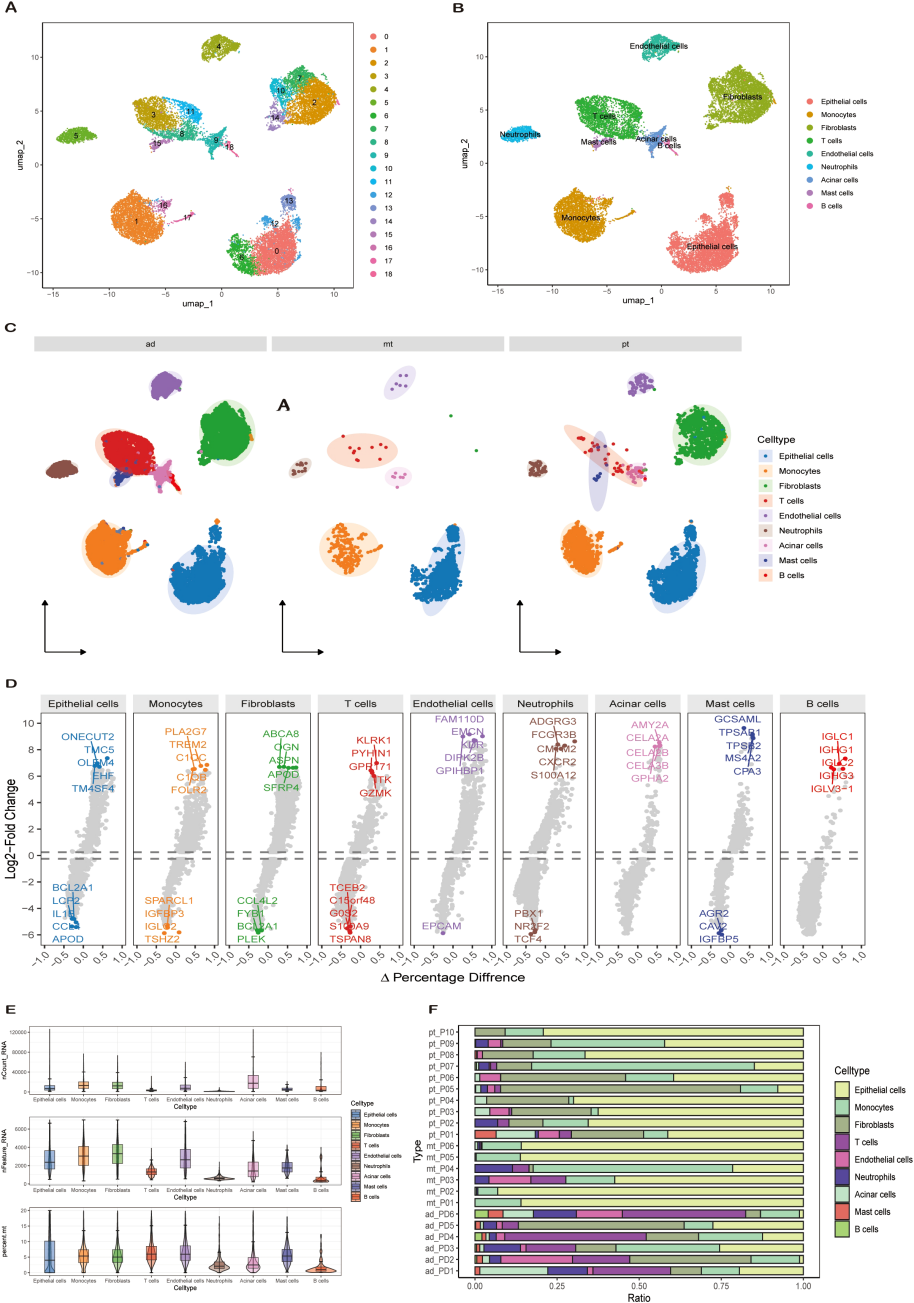
Single-cell transcriptomic atlas of pancreatic cancer. **(A)** UMAP visualization of 19 transcriptionally distinct cell clusters obtained through unsupervised clustering (resolution = 0.8). **(B)** Annotation and validation of nine major cell lineages based on canonical marker genes. **(C)** Integrated visualization of multiple samples following batch-effect correction, with 95% confidence ellipses indicating cell type distribution. **(D)** Heatmap displaying expression patterns of key marker genes across identified cell types. **(E)** Distribution of quantitative molecular features (e.g., nCount_RNA, nFeature_RNA) across cellular populations. **(F)** Comparative analysis of cellular composition heterogeneity among samples, assessed by Kruskal-Wallis test (P < 0.05).

We constructed a comprehensive cellular atlas of pancreatic cancer by integrating scRNA-seq data from 22 patients in the GSE154778 and GSE212966 datasets, encompassing 10 primary tumors, 6 adjacent normal tissues, and 6 metastatic lesions. A standardized Seurat v5 workflow was employed, selecting the first 11 principal components (PCs) for downstream analysis based on cumulative variance and JackStraw significance (P < 1×10^-5^). Harmony integration effectively corrected for batch effects, and Leiden clustering (resolution=0.8) identified 19 transcriptionally distinct subpopulations (**Figure 1A**). These were consolidated into nine major cell types through automated (SingleR) and manual annotation using canonical markers (**Figure 1B**). Visualization via UMAP confirmed consistent spatial organization of cell types across datasets (**Figure 1C**), which was further validated by the expression of established marker genes (**Figure 1D**). Analysis of transcriptional activity identified epithelial cells, monocytes, and fibroblasts as the most feature-rich populations within the TME (**Figure 1E**). Comparative cellular quantification revealed significant compositional shifts: adjacent normal tissues were enriched for T cells (21.85% ± 4% higher infiltration, P < 0.001), whereas tumor tissues showed a marked expansion of epithelial cells (43% ± 10% enrichment, P < 0.01). Primary tumors further exhibited increased fibroblast infiltration compared to metastatic lesions (24% ± 10%, P < 0.01) (**Figure 1F**).

### 3.2 Integrative genetics and network analysis identifies pro-tumorigenic modules

**Figure 2:**
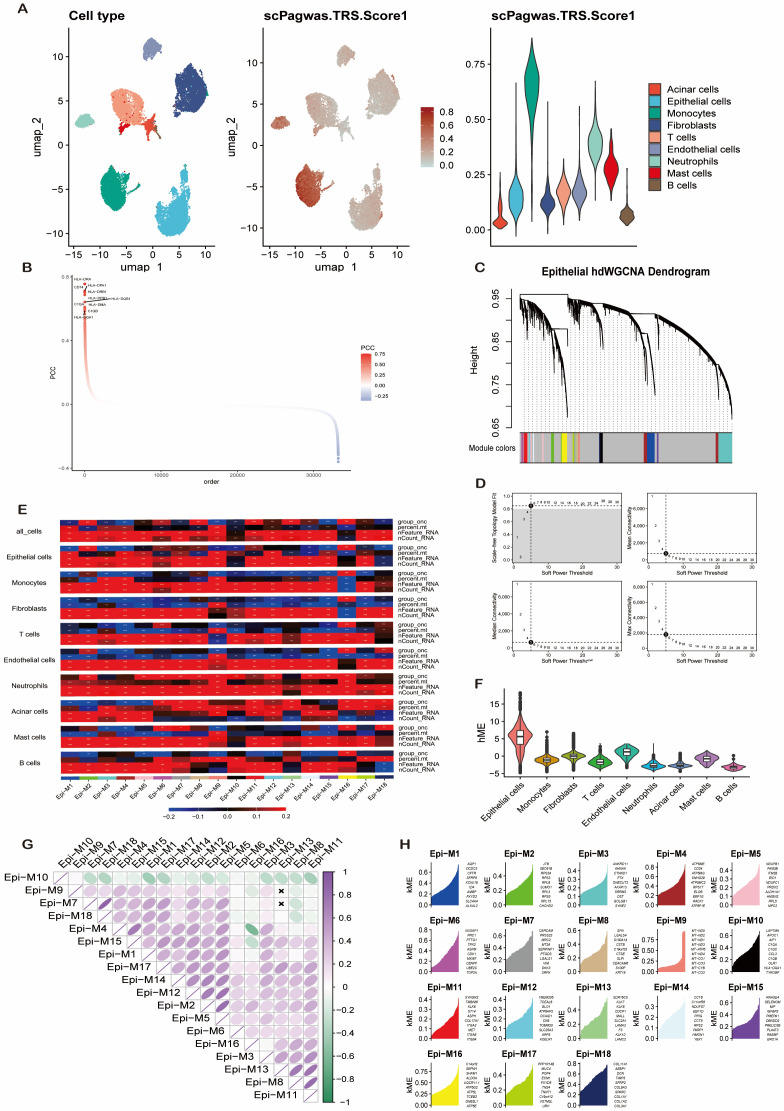
Integrative genetic and co-expression network analysis identifies pancreatic cancer-associated modules. **(A)** Cell type-specific trait relevance score (TRS) distribution derived from scPagwas algorithm. **(B)** Phenotype-correlation matrix illustrating Pearson correlation coefficients (PCC) between cellular TRS and pancreatic cancer traits; red intensity denotes positive association strength. **(C)** Hierarchical clustering dendrogram of co-expression genes from hdWGCNA, with dynamically color-coded modules (min module size = 30, cut height = 0.25). **(D)** Analysis of network topology for soft-thresholding power selection in hdWGCNA (scale-free topology fit index R² > 0.85). **(E)** Module-trait relationship heatmap quantifying associations between co-expression modules and clinical phenotypes. **(F)** Protein-protein interaction network visualization of hub genes within hdWGCNA Module 11. **(G)** Eigengene correlation network depicting functional relationships among key co-expression modules. **(H)** Violin plots showing expression patterns of epithelial cell-specific marker genes identified from characteristic hdWGCNA modules.

Application of the scPagwas algorithm to map genetic associations onto single-cell data revealed significant heterogeneity in Trait Relevance Scores (TRS) across cell types. Monocytes displayed the highest TRS (Kruskal-Wallis test, P < 0.01), while B cells and acinar cells showed the lowest (P < 0.05) (**Figure 2A**). The distribution of gene-trait association strengths (Pearson’s R) followed a bimodal pattern (**Figure 2B**). To identify robust genetic candidates, we filtered for genes with |R| > 0.1 and FDR-adjusted P < 0.05, yielding 669 candidates for further analysis.

Parallelly, hdWGCNA constructed a weighted co-expression network from the single-cell data. A soft threshold power of 5 was selected to achieve a scale-free topology (R² > 0.85) (**Figure 2D**), resulting in 18 distinct co-expression modules (**Figure 2C**). Module-trait correlation analysis identified three modules—Module 8 (R = 0.37, P<0.05), Module 11 (R = 0.33, P<0.05), and Module 16 (R = 0.95, P<0.05)—as being strongly positively correlated with pancreatic cancer traits (**Figure 2E**). Notably, epithelial cells exhibited the highest hub module eigengene (hME) scores for Module 11, suggesting a central role in tumor-promoting processes like EMT (**Figure 2F**). Strong interconnectivity was observed between Modules 8 and 11 (**Figure 2G**), and the expression patterns of trait-associated genes from these high-priority modules were visualized across epithelial subpopulations (**Figure 2H**).

### 3.3 A multi-method machine learning framework for gene prioritization

**Figure 3:**
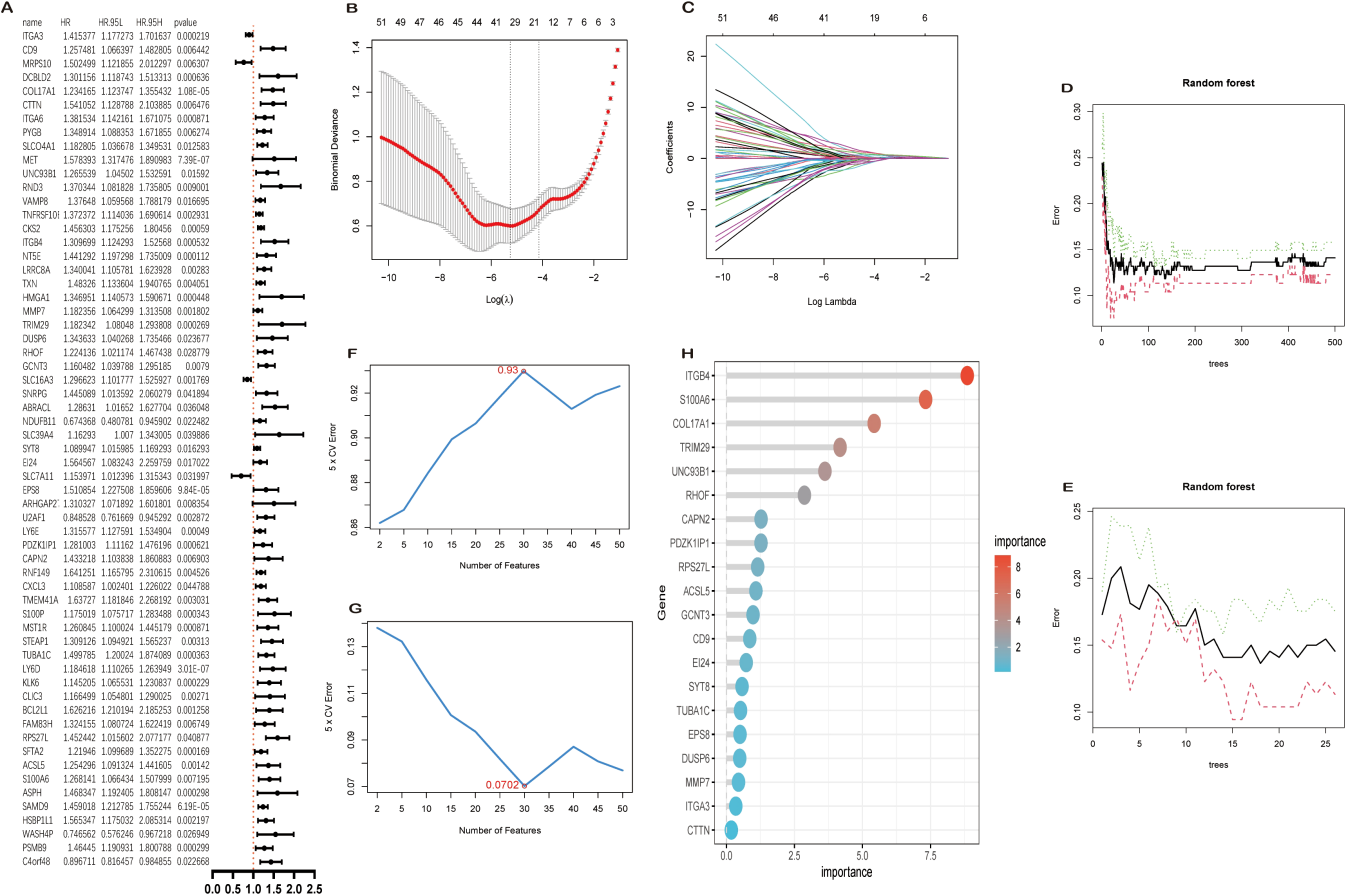
Machine learning-based screening of prognostic gene signatures. **(A)** Forest plot of univariate Cox regression analysis for prognosis-associated genes (P < 0.05), presenting hazard ratios (HR) with 95% confidence intervals. **(B)** Cross-validation error curve for LASSO regression (10-fold) with optimal lambda (λ) selection. **(C)** LASSO coefficient profile diagram showing feature selection paths across regularization penalties. **(D)** Random forest out-of-bag (OOB) error convergence across 500 decision trees. **(E)** OOB error rate of the optimal random forest submodel comprising 26 trees. **(F, G)** Accuracy metrics and error curves from support vector machine recursive feature elimination (SVM-RFE) algorithm. **(H)** Variable importance ranking of the top 20 genes from random forest analysis.

A candidate pool of 320 genes was established from the intersection of 1,381 epithelial cell-enriched differentially expressed genes (DEGs) and 719 genes derived from hdWGCNA. Univariate Cox regression analysis against TCGA survival data refined this set to 61 genes significantly associated with patient prognosis (P < 0.05), designated as “key genes” (**Figure 3A**).

This gene set was further refined using a tripartite machine learning approach applied to bulk transcriptomic datasets (GSE28735, GSE62452). LASSO regression identified 34 non-redundant features, from which the top 20 were selected (**Figures 3B, C**). Similarly, Random Forest (**Figures 3D, E, H**) and Support Vector Machine with Recursive Feature Elimination (SVM-RFE; **Figures 3F, G**) each provided a ranked list of the top 20 genes based on feature importance.

### 3.4 UNC93B1 emerges as a core diagnostic and prognostic determinant

**Figure 4:**
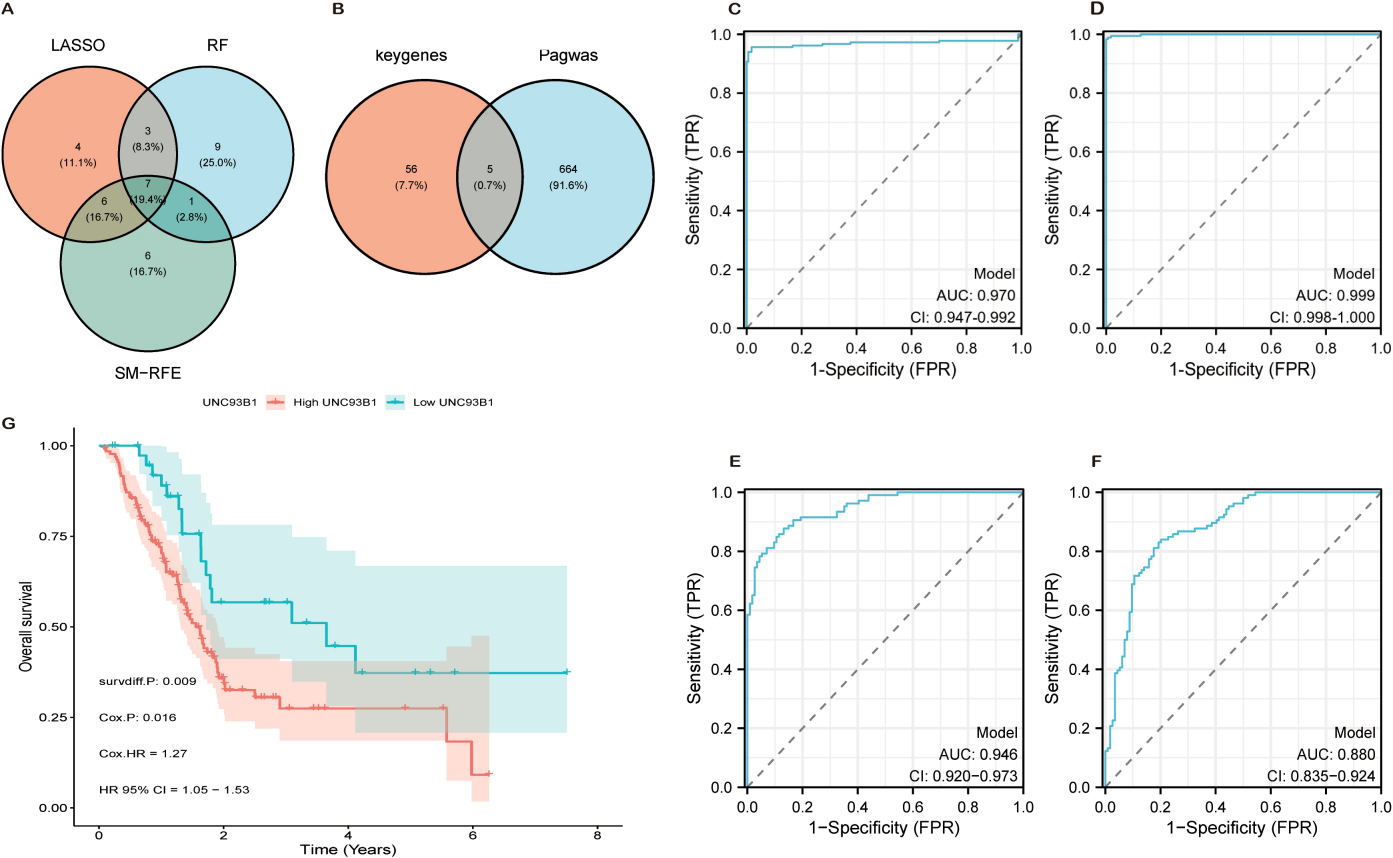
Diagnostic validation and clinical prioritization of UNC93B1. **(A)** Venn diagram illustrating the intersection of candidate genes identified by three machine learning algorithms (n = 7 overlapping genes). **(B)** Overlap between scPagwas-derived genes and machine learning-identified diagnostic candidates (n = 5 genes). **(C, D)** Receiver operating characteristic (ROC) curves and area under the curve (AUC) values evaluating diagnostic performance of MLDS and PADS in TCGA-GTEx cohort. **(E, F)** Validation of diagnostic efficacy for MLDS and PADS in independent bulk RNA-seq datasets. **(G)** Kaplan-Meier survival analysis comparing overall survival between UNC93B1 high- and low-expression groups in TCGA cohort (log-rank P = 0.009).

The intersection of the top 20 genes from the three machine learning algorithms defined a 7-gene Machine Learning-Derived Signature (MLDS): S100A6, COL17A1, CTTN, UNC93B1, CD9, TUBA1C, and ITGB4 (**Figure 4A**). MLDS demonstrated robust diagnostic power, achieving an Area Under the Curve (AUC) of 0.97 in the TCGAxGTEx cohort and 0.946 in independent bulk datasets (**Figures 4C, E**).

Further integration with scPagwas results identified a 5-gene scPagwas-Associated Diagnostic Signature (PADS): UNC93B1, VAMP8, SLC16A3, RNF149, and CXCL3 (**Figure 4B**). PADS showed exceptional performance in TCGAxGTEx (AUC = 0.999) and good performance in bulk datasets (AUC = 0.88) (**Figures 4D, F**). Crucially, UNC93B1 was the sole gene common to both MLDS and PADS, highlighting its unique position as a consensus high-priority candidate. Kaplan-Meier analysis confirmed its prognostic value, with high UNC93B1 expression correlating with significantly worse overall survival in the TCGA-PAAD cohort (P = 0.009) (**Figure 4G**).

### 3.5 Multi-omic profiling establishes UNC93B1 as a PDAC-associated oncogene

**Figure 5:**
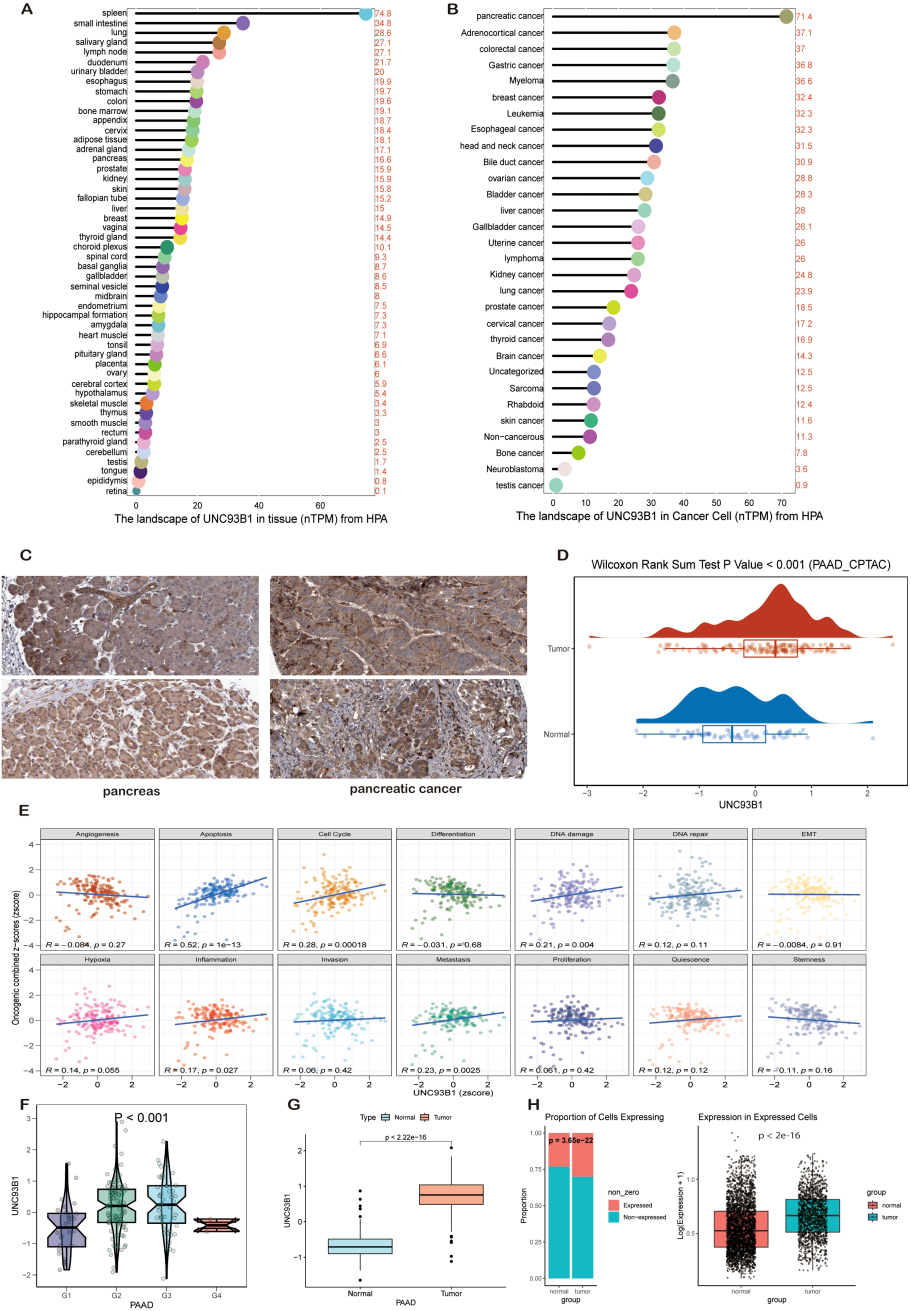
Multi-omics characterization of UNC93B1 in pancreatic cancer. **(A)** Pan-tissue expression profile of UNC93B1 across normal human tissues, presented as normalized transcripts per million (nTPM). **(B)** Pan-cancer expression landscape of UNC93B1 across various cancer cell lines, highlighting elevated expression in pancreatic cancer. **(C)** Immunohistochemical **(IHC)** validation of UNC93B1 protein localization in pancreatic tissues from Human Protein Atlas (tumor samples: HPA042803; normal samples: HPA038717). **(D)** Comparative proteomic analysis of UNC93B1 expression in CPTAC cohort, showing differential abundance between tumor and adjacent normal tissues (Wilcoxon test, P < 0.05). **(E)** Functional state correlation analysis between UNC93B1 expression z-scores and combined z-scores of cancer-related phenotypes, annotated with Pearson correlation coefficients (R) and adjusted P-values. **(F)** Association between UNC93B1 expression levels and pathological tumor grade in TCGA-PAAD cohort (Kruskal-Wallis test, P < 0.001). **(G)** Differential UNC93B1 expression between pancreatic tumor and normal tissues in TCGA-GTEx dataset (Wilcoxon test, P < 0.001). **(H)** Single-cell RNA-seq validation of elevated UNC93B1 expression in pancreatic tumor cells from GSE154778 and GSE212966 datasets (Wilcoxon test, P < 0.001).

Comprehensive pan-tissue analysis delineated a heterogeneous expression profile for UNC93B1, with peak transcript levels detected in the spleen, small intestine, and lung. In contrast, its expression in the normal pancreas was comparatively modest, ranking 16th among all tissues surveyed (**Figure 5A**). A striking exception was observed in pancreatic cancer, where UNC93B1 expression was markedly elevated across cell lines, exceeding levels found in all other carcinoma types profiled (**Figure 5B**). This tumor-specific overexpression was consistently validated at the protein level. Immunohistochemical (IHC) analysis of specimens from the Human Protein Atlas (HPA) revealed intense UNC93B1 immunoreactivity in pancreatic ductal adenocarcinoma (PDAC) samples (HPA042803; Patients 3851, 4079), in stark contrast to the faint staining observed in normal pancreatic tissues (HPA038717; Patients 1647, 2329) (**Figure 5C**). Complementary proteomic data from the Clinical Proteomic Tumor Analysis Consortium (CPTAC) further corroborated a significant increase in UNC93B1 protein abundance in PDAC compared to non-neoplastic counterparts (**Figure 5D**).

Transcriptomic interrogation at single-cell resolution (GSE154778, GSE212966) and analysis of TCGA pan-cancer data (**Figures 5G, H**) uniformly demonstrated a significant elevation in both the prevalence of UNC93B1-expressing cells and the transcript abundance per cell within pancreatic tumors, effectively overcoming the inherent sparsity of single-cell data. Clinically, elevated UNC93B1 expression exhibited a strong positive correlation with advanced pathological grade (P < 0.001), being significantly higher in G2/G3 tumors compared to G1 (**Figure 5F**). Functional characterization using CancerSEA associated high UNC93B1 expression with several hallmarks of malignancy, including apoptosis (R = 0.52, P<0.001), cell cycle progression (R = 0.28, P = 0.003), DNA damage (R = 0.21, P = 0.018), and metastasis (R = 0.23, P = 0.011) (**Figure 5I**). Spatial transcriptomics provided final confirmation, localizing high UNC93B1 expression predominantly within tumor regions, with minimal signal in adjacent non-tumor areas, suggesting a spatially restricted role in pancreatic oncogenesis (**Supplementary Figure 1**).

### 3.6 Pseudotime trajectory analysis implicates UNC93B1 in disease progression

**Figure 6:**
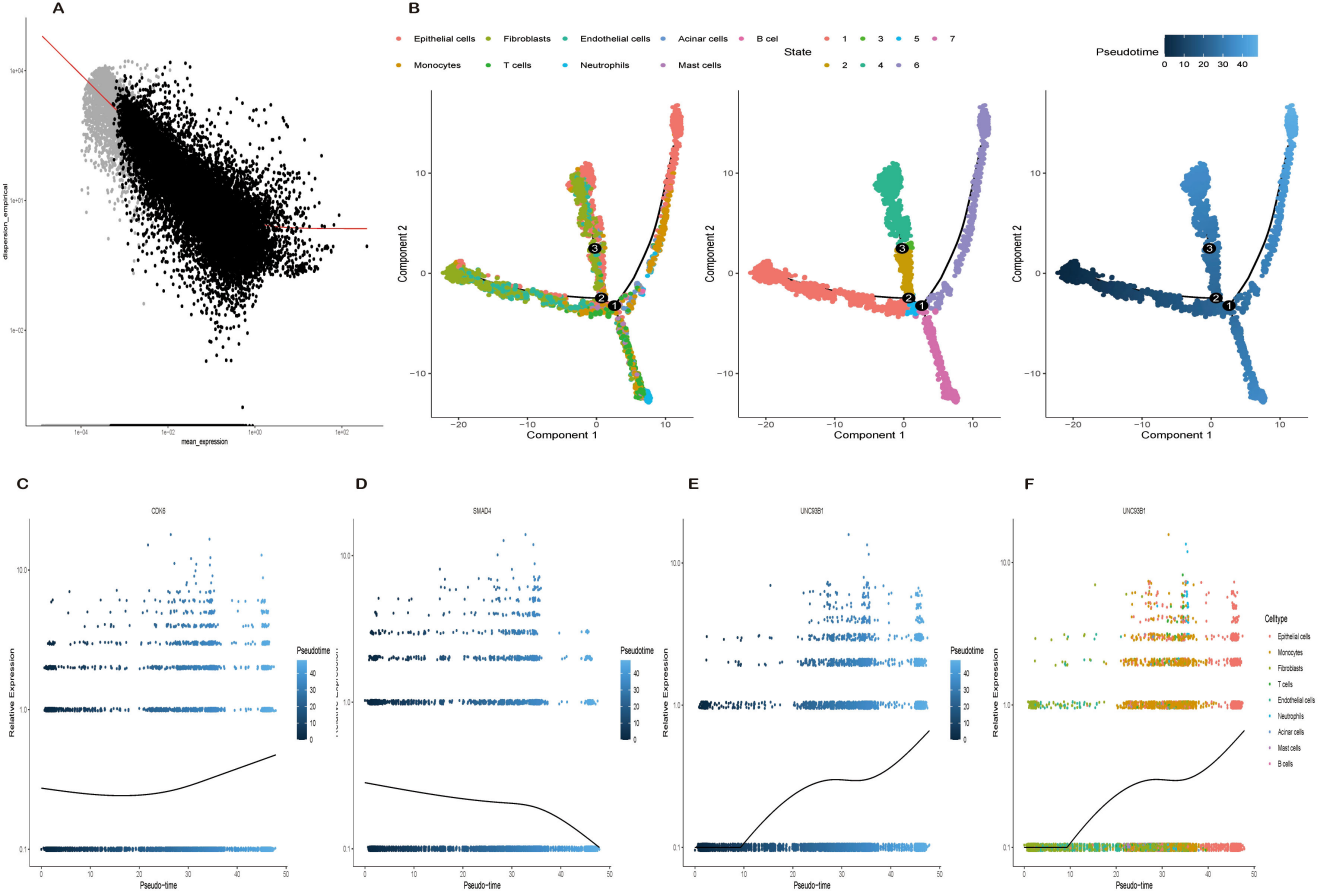
Single-cell trajectory analysis reveals dynamic expression patterns during disease progression. **(A)** Schematic of trajectory-driven gene screening methodology for identifying regulators of cell-state transitions. **(B)** Reconstructed pseudotemporal trajectory showing multi-branch differentiation paths and seven distinct cell states, ordered by developmental progression. **(C)** Pseudotime-dependent expression kinetics of CDK6, demonstrating progressive upregulation along trajectory. **(D)** Pseudotime-dependent downregulation of SMAD4, consistent with its known role in TGF-β signaling suppression during tumor progression. **(E)** Pseudotemporal expression pattern of UNC93B1, showing marked upregulation correlating with advanced differentiation states. **(F)** Cellular cluster heterogeneity in UNC93B1 expression across subpopulations.

Leveraging trajectory-driven gene screening (**Figure 6A**), we employed pseudotemporal analysis to reconstruct the progression of pancreatic cancer cells. This analysis revealed a multi-branched differentiation topology characterized by three branch points and seven distinct cellular states (States 1-7; **Figure 6B**). The trajectory originated from a low-differentiation state (State 1) and culminated in terminal states (States 5-7) associated with highly metastatic and dedifferentiated phenotypes. UNC93B1 expression demonstrated a clear pseudotime-dependent increase (**Figure 6E**), positioning it as a potential positive regulator of tumor progression, a pattern further corroborated by its distinct expression across cellular subtypes (**Figure 6F**).

For context, we also analyzed the dynamics of established PDAC genes. CDK6, a cell cycle regulator that promotes G1/S transition via Cyclin D-dependent Rb phosphorylation (32), showed a progressive upregulation along pseudotime (**Figure 6C**), consistent with its known role in PDAC. Conversely, SMAD4, a central transcriptional mediator of TGF-β signaling involved in growth control and apoptosis (33), was progressively downregulated (**Figure 6D**), aligning with the loss of TGF-β tumor-suppressive responses in advanced disease. While pseudotime analysis models transcriptional similarity rather than absolute time, these findings collectively suggest that UNC93B1 overexpression drives pancreatic cancer cells toward more proliferative and aggressive states.

### 3.7 Pathway enrichment analysis converges on the cGAS-STING pathway

**Figure 7:**
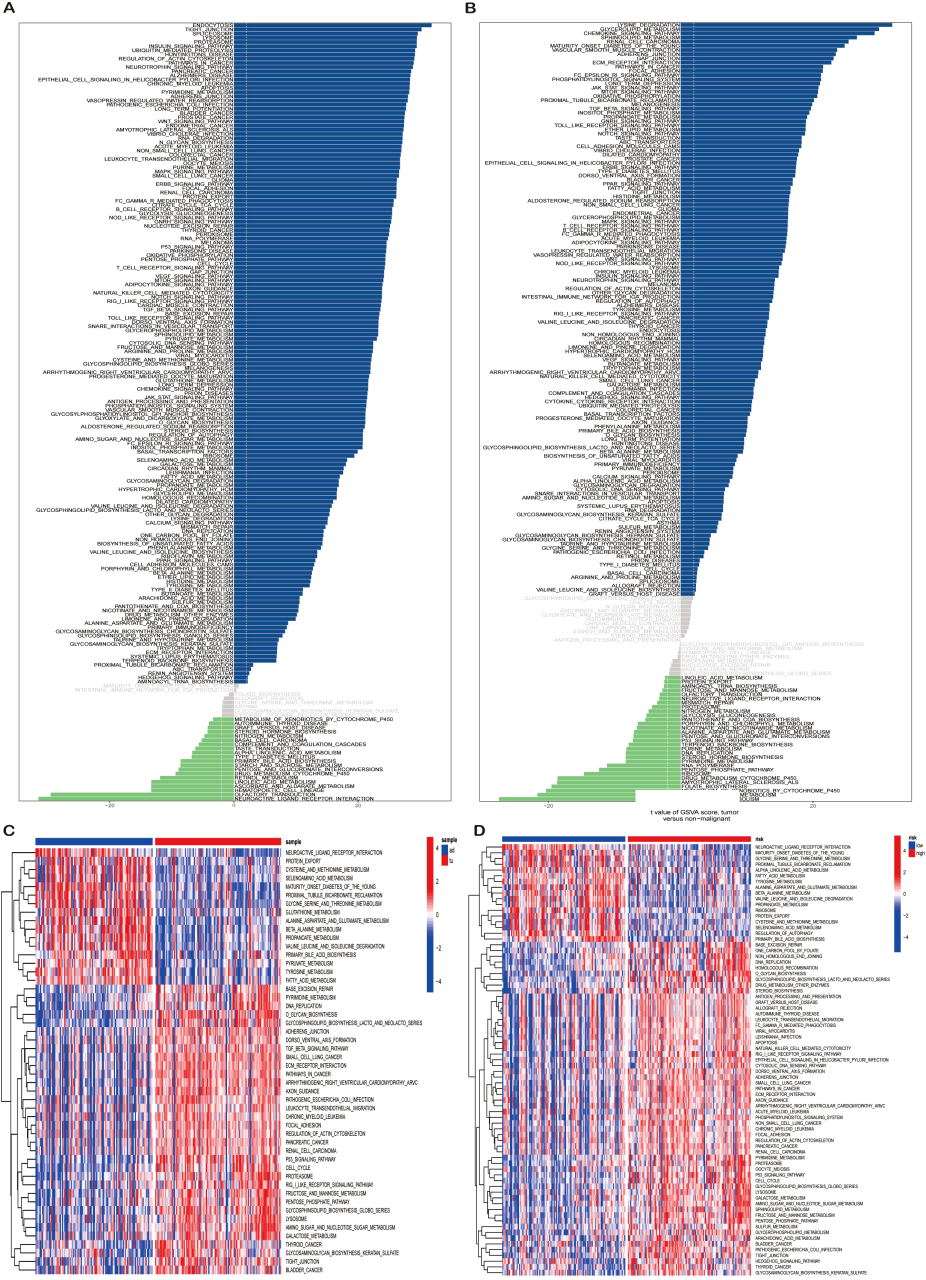
Pathway enrichment analysis using gene set variation analysis (GSVA). **(A)** GSVA of single-cell datasets (GSE154778, GSE212966) comparing pathway enrichment between tumor and normal tissues. **(B)** GSVA comparison of pathway activities between UNC93B1 high- and low-expression subgroups in single-cell datasets. **(C)** Bulk RNA-seq datasets (GSE28735, GSE62452) analyzed for tumor-normal differential pathway enrichment. **(D)** Parallel GSVA of UNC93B1 expression subgroups in bulk transcriptomic datasets.

To elucidate the functional mechanisms of UNC93B1, we performed a systematic Gene Set Variation Analysis (GSVA) across multiple datasets. In single-cell data (GSE154778, GSE212966), GSVA compared tumor vs. normal tissues (**Figure 7A**) and UNC93B1-high vs. UNC93B1-low subgroups (**Figure 7B**). A parallel analysis in bulk transcriptomic datasets (GSE28735, GSE62452) evaluated the same contrasts (**Figures 7C, D**). A cross-dataset intersection of significantly enriched pathways consistently identified the RIG-I-like receptor (RLR) signaling pathway, the cytosolic DNA-sensing (cGAS-STING) pathway, and the Hedgehog (Hh) signaling pathway, suggesting their core involvement in the UNC93B1-associated regulatory network.

Critically, a review of existing literature revealed that among these pathways, UNC93B1 has a previously established, direct role in negatively regulating the cGAS-STING pathway by mediating its autophagic-lysosomal degradation (25),. In contrast, its functional links to the RLR and Hh pathways in pancreatic cancer remain unreported. Therefore, integrating our consistent bioinformatic enrichment of the cGAS-STING pathway in UNC93B1-high contexts, we hypothesize that UNC93B1 exerts its oncogenic effects in PDAC primarily through the suppression of cGAS-STING-mediated innate immunity.

### 3.8 UNC93B1 remodels the intercellular communication network

**Figure 8:**
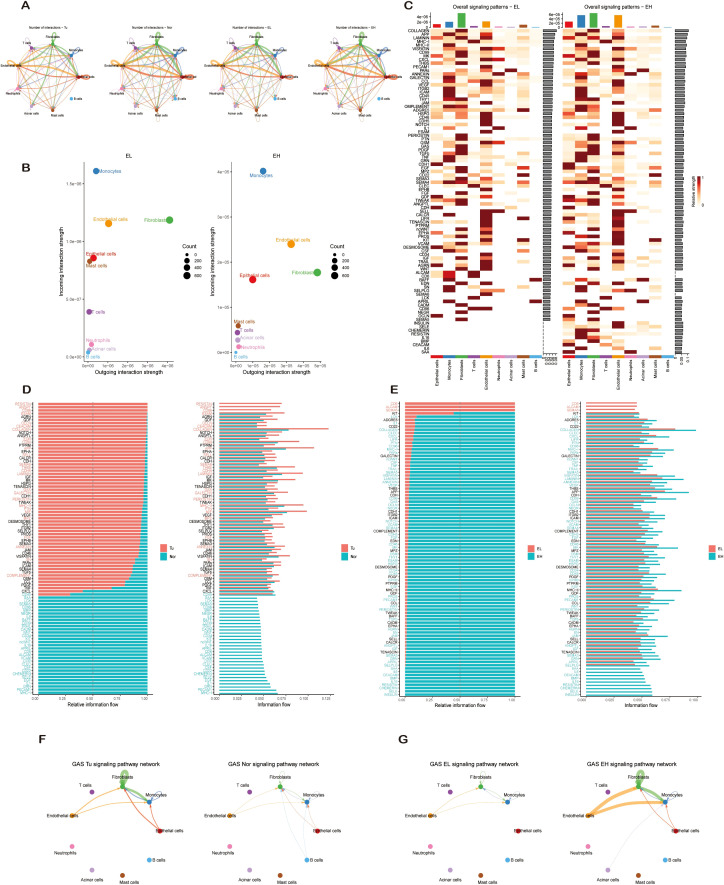
Intercellular communication network analysis in tumor microenvironment. **(A)** Comprehensive cell-cell interaction networks comparing tumor versus normal microenvironments and UNC93B1-dependent signaling contexts. **(B)** Differential intensity analysis of outgoing and incoming signaling patterns across UNC93B1-dependent cell clusters. **(C)** Heatmap visualization of UNC93B1-dependent outgoing and incoming signaling patterns. **(D)** Comparative visualization of pathway activity differences between tumor and normal microenvironments. **(E)** Differential pathway activity analysis in UNC93B1-dependent subgroups. **(F)** cGAS-STING signaling interaction networks in tumor versus normal microenvironments. **(G)** UNC93B1-dependent cGAS-STING signaling interaction networks.

Cell-cell communication analysis revealed distinct interaction patterns. Non-tumor tissues exhibited greater interaction diversity, whereas UNC93B1-high groups demonstrated enhanced interaction complexity (**Figure 8A**). Pathway activity analysis indicated that signaling strength was significantly higher in tumor tissues versus normal, and in UNC93B1-high versus UNC93B1-low subgroups (**Figures 8D, E**). This paints a nuanced picture: non-tumor tissues engage in more diverse, but weaker interactions, while UNC93B1-high groups exhibit more focused yet potent communication.

Analysis of signaling roles identified monocytes as the dominant signal receivers (highest incoming degree), consistent with their high genetic trait relevance scores from prior analysis and their role as antigen-presenting cells (**Figures 8B, C**). Fibroblasts, in contrast, served as the primary signal senders (highest outgoing degree). Notably, epithelial cells in UNC93B1-high groups showed a shift towards increased outgoing signaling activity, coupled with a mild but specific activation of the GAS pathway (**Figures 8F, G**), suggesting UNC93B1 reshapes the TME by enhancing pro-tumorigenic output from epithelial cells. Interestingly, the Hh and RLR pathways, identified by GSVA, did not show significant alterations in their cell-cell communication dynamics.

### 3.9 Generation and validation of a stable UNC93B1-knockdown model

**Figure 9:**
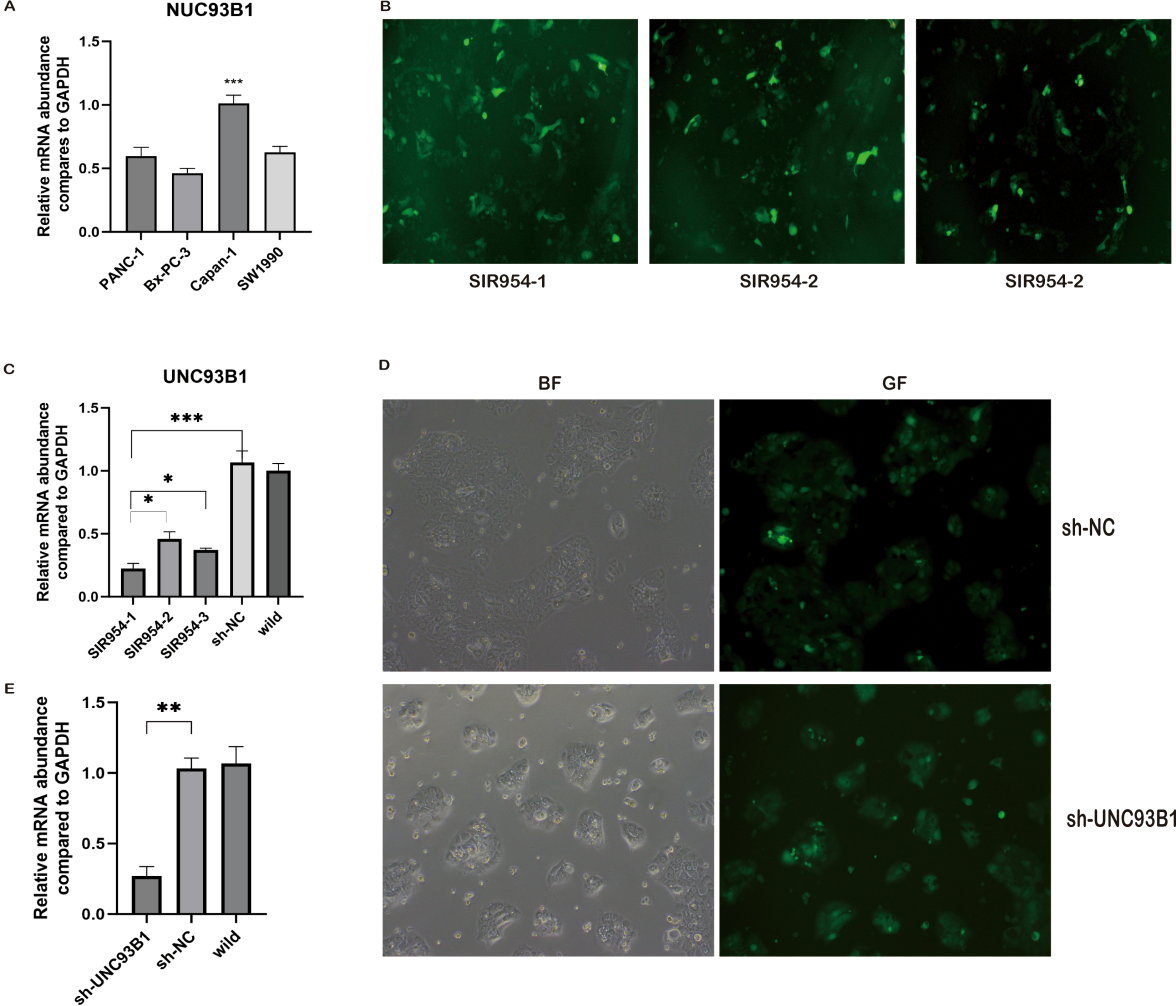
Establishment and validation of stable UNC93B1-knockdown cell models. **(A)** Quantitative PCR (qPCR) analysis of basal UNC93B1 expression across four pancreatic cancer cell lines. **(B)** Fluorescence microscopy images demonstrating transduction efficiency in Capan-1 cells using three independent shRNA constructs (SIR954-1, -2, -3). **(C)** qPCR validation of UNC93B1 knockdown efficiency following lentiviral transduction. **(D)** Representative brightfield (BF) and green fluorescence (GF) micrographs comparing sh-UNC93B1 and scramble shRNA control (sh-NC) stable cell pools. **(E)** qPCR analysis confirming sustained UNC93B1 suppression throughout serial cell passages. (Abbreviations: BF, brightfield; GF, green fluorescence; sh-NC, scramble short hairpin RNA negative control. *P < 0.05, **P < 0.01, ***P < 0.001).

To establish a robust system for functional interrogation, we first quantified UNC93B1 mRNA expression across a panel of PDAC cell lines (Panc-1, BxPC-3, Capan-1, SW1990) via qPCR (**Figure 9A**). Capan-1 cells demonstrated the highest endogenous UNC93B1 expression (1.01 ± 0.16; P < 0.001 vs. others) and were thus selected for knockdown studies.

We designed three distinct lentiviral shRNAs targeting UNC93B1. Transduction of Capan-1 cells achieved high efficiency (>70% GFP-positive cells; **Figure 9B**). qPCR confirmed that all three shRNAs significantly suppressed UNC93B1 mRNA (P < 0.001), with SIR954–1 proving most effective (0.20 ± 0.02 vs. sh-NC 1.00 ± 0.11; P < 0.05 vs. other shRNAs) (**Figure 9C**). The SIR954–1 construct was used to generate a stable polyclonal knockdown population (sh-UNC93B1) under puromycin selection. qPCR validation across five consecutive passages confirmed sustained knockdown, with an average reduction of 82% compared to the non-targeting control (sh-NC) (P < 0.01; **Figures 9D, E**).

### 3.10 UNC93B1 knockdown suppresses malignant phenotypes *In vitro*

**Figure 10:**
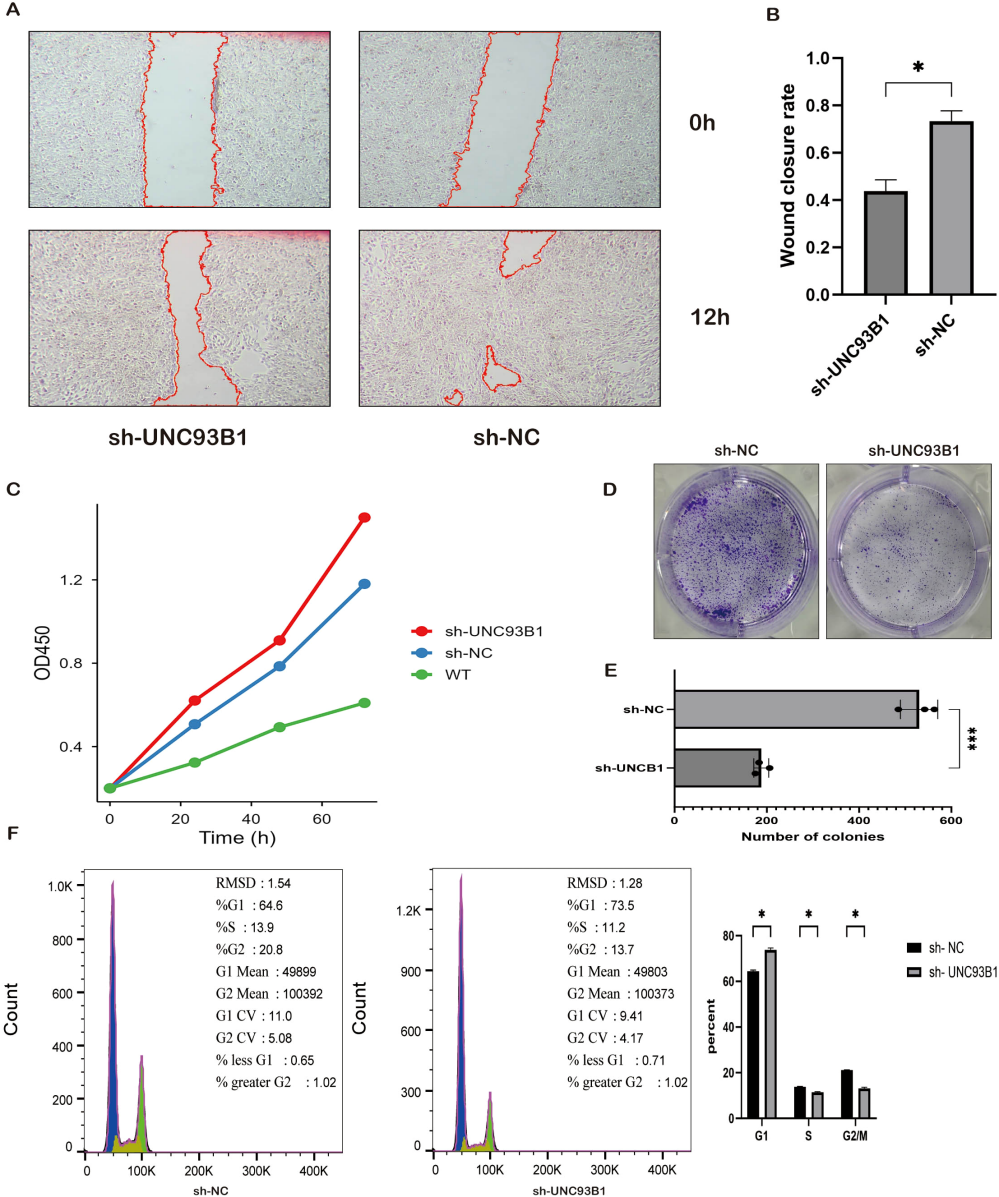
Functional characterization of UNC93B1-knockdown in pancreatic cancer cells. **(A, B)** Scratch wound healing assay quantifying migratory capacity of control versus UNC93B1-knockdown cells at 0, 24, and 48 hours. **(C)** CCK-8 proliferation assay measuring cellular viability over 5 days. **(D, E)** Colony formation assay assessing clonogenic potential following UNC93B1 depletion. **(F)** Cell cycle distribution analysis by flow cytometry with propidium iodide (PI) staining, showing G1 phase arrest in knockdown cells. (*P < 0.05, **P < 0.01, ***P < 0.001).

Functional characterization of the sh-UNC93B1 model revealed a profound impairment in oncogenic behaviors. Wound healing assays showed a significant reduction in migratory capacity, with a 29.6% ± 6% decrease in wound closure at 12 hours post-scratch (P < 0.05; **Figures 10A, B**). Proliferation, as measured by CCK-8 assay, was significantly attenuated in sh-UNC93B1 cells at 72 hours compared to both sh-NC and wild-type controls (ΔOD450 = 0.18 and 0.22, respectively; P < 0.05), with no difference between the latter two groups (**Figure 10C**). Clonogenic survival was also severely compromised, with sh-UNC93B1 cells forming significantly fewer (mean reduction: 342 ± 25 colonies, P < 0.01) and smaller colonies (0.43 ± 0.05 mm vs. 0.82 ± 0.07 mm, P < 0.01) (**Figures 10D, E**).

Transwell assay demonstrated a significant impairment in the invasive migration capacity of sh-UNC93B1 cells. Compared to the sh-NC control, knockdown of UNC93B1 resulted in a marked reduction in the number of cells that migrated through the Matrigel-coated membrane (P < 0.01; **Figures 10A, B**). This finding was consistent with the results obtained from the wound healing assay, further confirming the critical role of UNC93B1 in promoting the migratory and invasive potential of PDAC cells.

Cell cycle analysis via PI-based flow cytometry (**Figure 10F**) revealed high-quality data (G1/G2 peak ratios ~2.0, low G2 CV). UNC93B1 knockdown induced a significant G1-phase arrest, increasing the G1 population by 8.9% (73.5% vs. 64.6% in sh-NC; P < 0.05) while concurrently decreasing the proportions of cells in S-phase and G2/M-phase (P < 0.05), indicating a blockade in cell cycle progression.

### 3.11 Molecular profiling indicates cGAS-STING activation and EMT suppression

**Figure 11:**
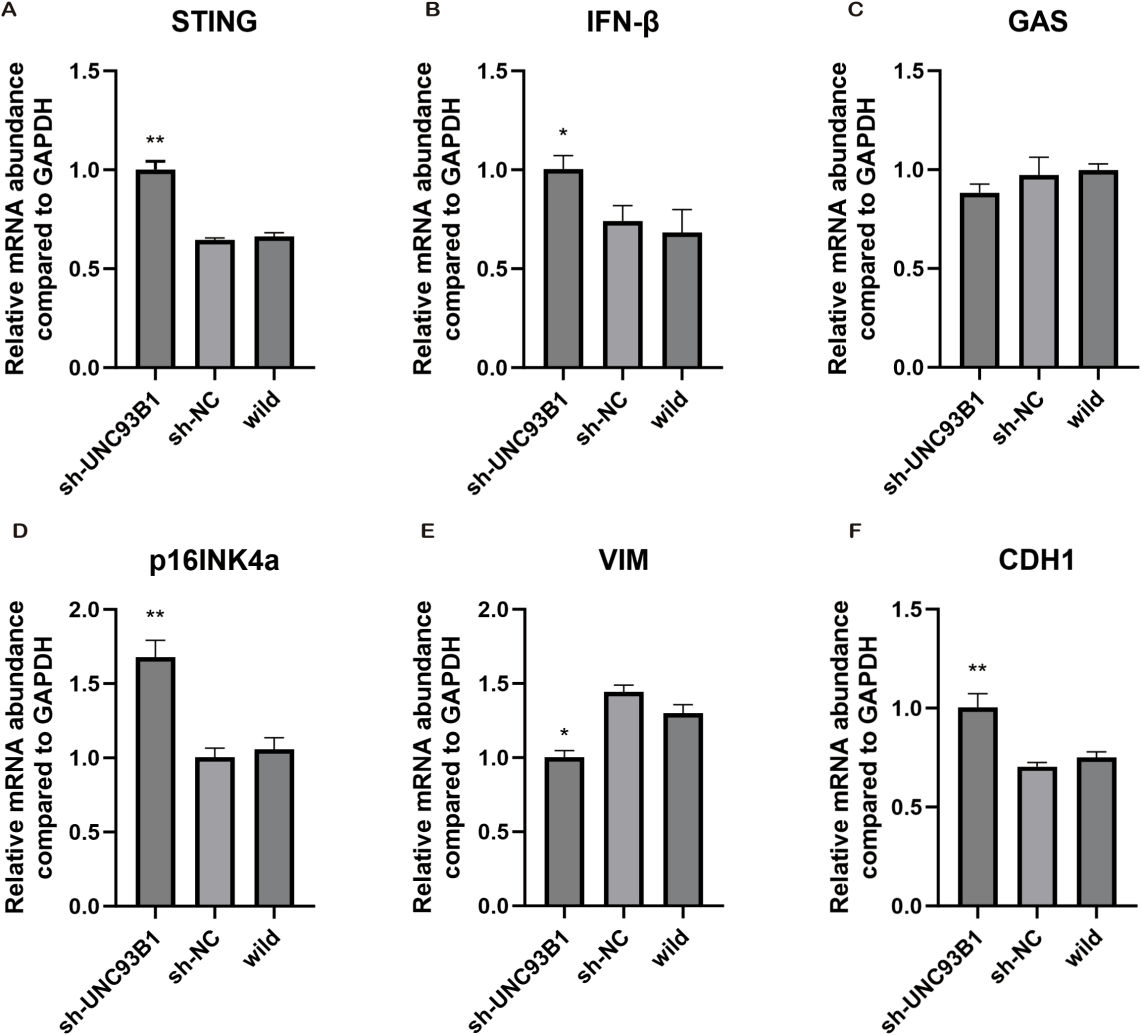
Molecular profiling of cGAS-STING pathway and associated phenotypes following UNC93B1 manipulation. **(A–F)** Quantitative PCR analysis of key genes in the cGAS-STING pathway (STING, IFN-β, cGAS), cellular senescence marker (p16INK4A), and epithelial-mesenchymal transition regulators (VIM, CDH1) following UNC93B1 knockdown. (*P < 0.05, **P < 0.01, ***P < 0.001).

To molecularly decipher the observed phenotypes, we analyzed the cGAS-STING pathway. qPCR analysis demonstrated that UNC93B1 knockdown significantly upregulated the mRNA expression of STING and its key downstream effector, IFN-β (**Figures 11A, B**), while cGAS transcript levels remained unchanged (**Figure 11C**). Furthermore, we observed a significant upregulation of the senescence-associated marker p16INK4a (P < 0.01; **Figure 11D**) (34). Concurrently, UNC93B1 silencing induced a shift towards a more epithelial state, characterized by downregulation of the mesenchymal marker VIM (vimentin) and upregulation of the epithelial marker CDH1 (E-cadherin) (**Figures 11E, F**), indicating an inhibition of the epithelial-mesenchymal transition (EMT).

### 3.12 *In vivo* validation confirms the tumor-promoting role of UNC93B1

**Figure 12:**
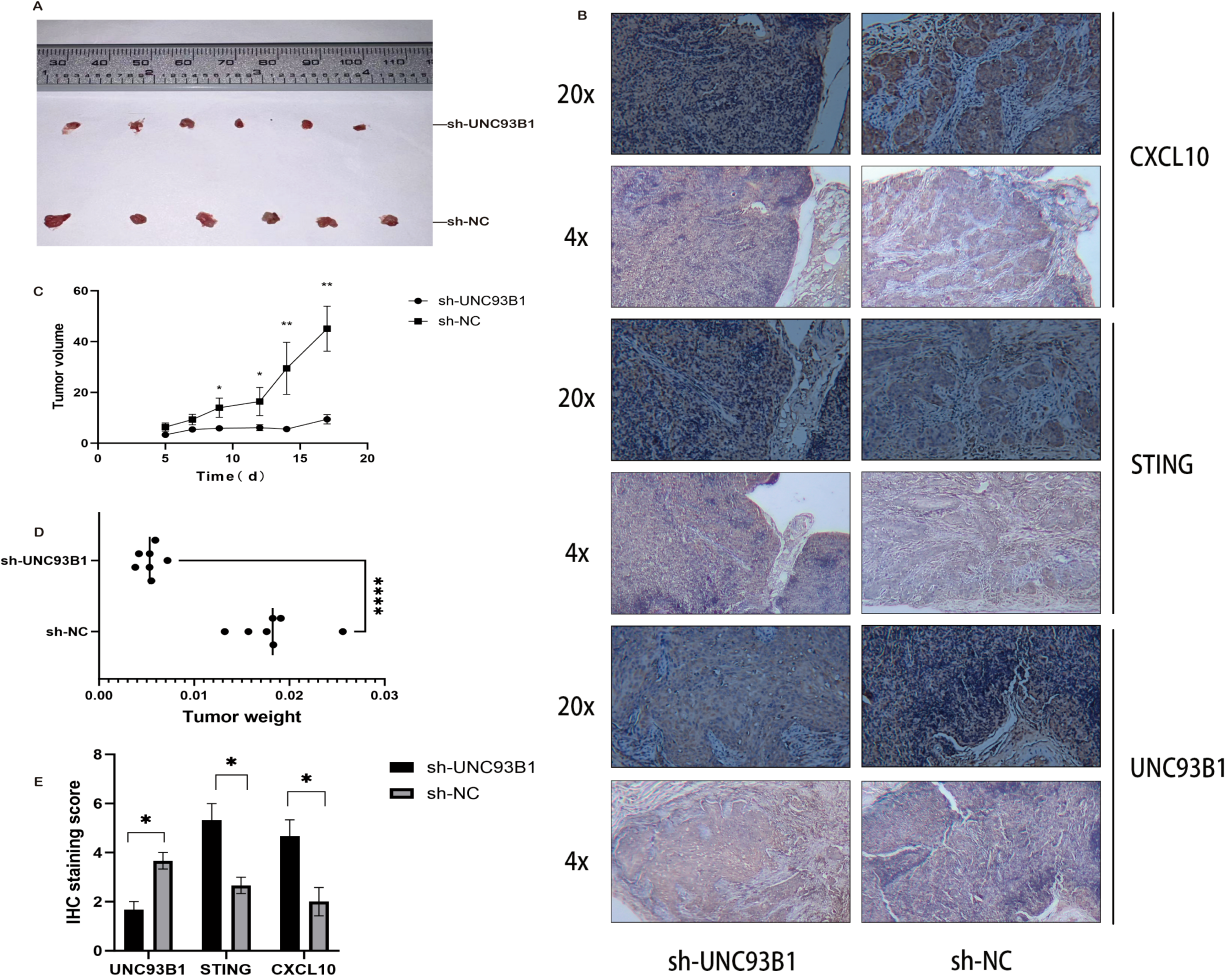
*In vivo* validation of UNC93B1 function in xenograft tumor models. **(A)** Representative photographs of excised tumors from nude mouse xenograft models (n = 6 per group). **(B)** Immunohistochemical (IHC) staining images of xenograft tumor sections. **(C)** Quantitative analysis of tumor volume progression throughout the experimental timeline. **(D)** Terminal tumor weight measurements at endpoint. **(E)** Statistical analysis of IHC scoring data. (*P < 0.05, **P < 0.01, ***P < 0.001, ****P < 0.0001).

The tumor-suppressive effect of UNC93B1 knockdown was conclusively validated in a nude mouse xenograft model. Tumors derived from sh-UNC93B1 cells exhibited significantly smaller volumes (P < 0.01; **Figures 12A, C**) and lower final weights (P < 0.01; **Figure 12D**) compared to the sh-NC control group. Notably, one mouse in the sh-UNC93B1 group failed to develop a palpable tumor, underscoring the role of UNC93B1 in tumor initiation.

IHC analysis of resected tumors provided mechanistic insight *in vivo*. Staining confirmed a significant reduction in UNC93B1-positive cells in the knockdown group (P < 0.05; **Figures 12B, E**). Conversely, and critically, we observed a marked increase in both the staining intensity and the proportion of cells positive for STING and its downstream chemokine CXCL10 (35) in the sh-UNC93B1 group (P < 0.05). These data provide direct *in vivo* evidence that UNC93B1 fosters PDAC growth by suppressing the anti-tumor STING pathway.

### 3.13 STING inhibition reverses the phenotypes induced by UNC93B1 depletion

**Figure 13:**
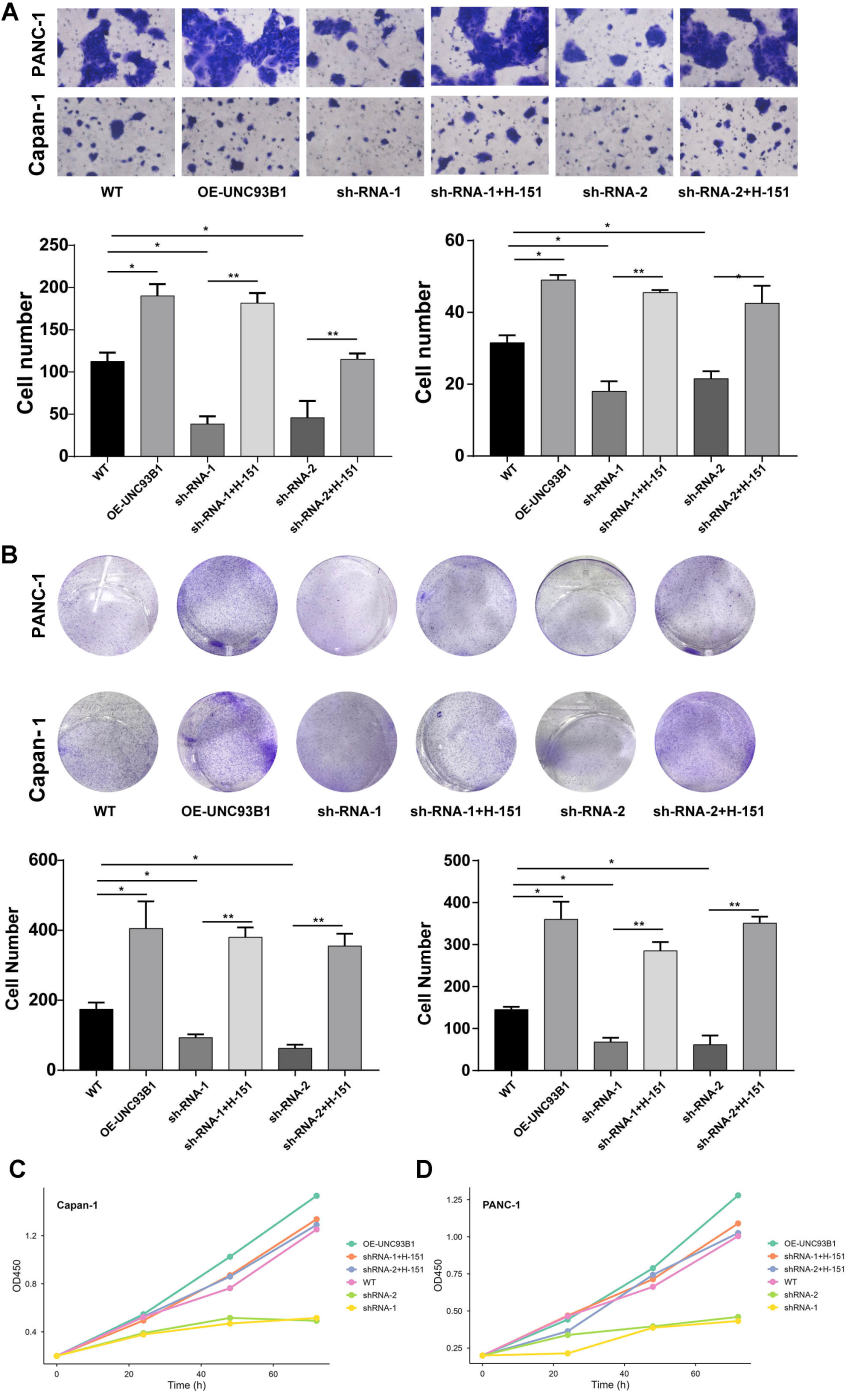
UNC93B1 knockdown phenotypes are rescued by STING inhibition. **(A)** Transwell migration assays in Capan-1 and PANC-1 cells under the indicated conditions. Representative images (upper panel) and quantitative analysis (lower panel) of migrated cells are shown. Data are presented as mean ± SD; n = 3 independent experiments. **(B)** Colony formation assays under the same conditions. Representative images of crystal violet-stained colonies (upper panel) and quantitative analysis of colony numbers (lower panel) are shown. **(C, D)** Cell proliferation kinetics measured by CCK-8 assay. Absorbance at 450 nm was recorded over 96 hours. Data are presented as mean ± SD; n = 6 replicates per group. Statistical significance was determined by one-way ANOVA with Tukey’s *post hoc* test. *P < 0.05, **P < 0.01, ***P < 0.001, ****P < 0.0001; ns, not significant.

To definitively determine whether the tumor-suppressive effects of UNC93B1 knockdown were mediated by hyperactivation of the cGAS-STING pathway, we performed genetic and pharmacological rescue experiments in two PDAC cell lines, Capan-1 and PANC-1. Using two independent shRNAs (sh-1 and sh-2), we confirmed that stable knockdown of UNC93B1 significantly impaired cell migration (**Figures 13A**) and clonogenic survival (**Figures 13B**), while its overexpression (OE-UNC93B1) enhanced these malignant properties. Critically, treatment with the specific STING inhibitor H-151 in the knockdown cells largely restored both migratory capacity and colony-forming ability to levels comparable with wild-type controls (**Figures 13A, B**). This rescue effect was consistently observed with both shRNAs, effectively ruling out off-target effects. Concordantly, the proliferative deficit caused by UNC93B1 knockdown, as measured by CCK-8 assay, was also markedly reversed upon STING inhibition (**Figures 13C, D**). These data provide direct causal evidence that the activation of the cGAS-STING pathway is the principal mechanism through which UNC93B1 loss exerts its tumor-suppressive effects in PDAC cells.

## 4 Discussion

The tumor immune microenvironment plays a fundamental role in the pathogenesis of pancreatic cancer (36–38). Single-cell transcriptional profiling has identified substantial T-cell infiltration in peri-tumoral regions, implying the presence of localized immune surveillance that may be initiated by early tumor antigen exposure (39). Conversely, neoplastic tissues demonstrated a marked decline in T-cell abundance, reflecting intensive immunoediting. This phenomenon may be driven by tumor-mediated suppression of effector T cells through upregulation of immune checkpoint molecules such as PD-L1 or recruitment of regulatory T cells (Tregs), collectively establishing immune-evasive niches (40). Such an immunosuppressive milieu, occurring alongside a high density of malignant epithelial cells, is likely instrumental in facilitating immune escape and disease progression.

Within primary tumors, expanded fibroblast populations are postulated to promote epithelial-mesenchymal transition (EMT) via paracrine factors including TGF-β and IL-6 (41, 42), thereby augmenting migratory and invasive properties. Interestingly, this process may be partially reversed through mesenchymal-epithelial transition (MET) within metastatic deposits, potentially aiding colonization at distant sites. The activated phenotype of fibroblasts in primary lesions underscores their central involvement in early pancreatic oncogenesis. Cancer-associated fibroblasts (CAFs) contribute to extracellular matrix (ECM) remodeling through secretion of fibronectin and collagen, generating dense physical barriers that limit chemotherapeutic penetration and foster treatment resistance. Moreover, CAF-derived chemokines such as CXCL12 recruit myeloid-derived suppressor cells, collectively reinforcing a pro-tumor inflammatory environment (43, 44).

Elevated transcriptional activity in monocytes implies their differentiation into tumor-associated macrophages (TAMs) (45). These TAMs frequently adopt an M2-polarized state, impairing T-cell antitumor functions through mechanisms involving arginase (ARG1) and indoleamine 2,3-dioxygenase (IDO), which deplete essential amino acids such as tryptophan (46). Increased glycolytic metabolism (evidenced by upregulation of HK2 and LDHA) in TAMs not only sustains their viability under hypoxic stress but also acidifies the extracellular milieu via lactate efflux, further stimulating angiogenesis and immune suppression.

The scPagwas algorithm bridges genome-wide association study (GWAS) summary statistics and single-cell transcriptomic data, enabling cross-scale mapping of genetic risk loci to cell type-specific pathways. This approach mitigates limitations related to tissue heterogeneity that often affect conventional Mendelian randomization (MR) analyses (22, 26). By computing trait-relevance scores (TRS), scPagwas quantifies the genetic contribution of distinct cellular subsets, pinpointing high-risk populations such as CAFs and their associated signaling cascades (e.g., EMT, inflammatory response) that correlate with clinical outcomes including chemotherapy resistance. Its principal innovation lies in dynamically integrating genetic variant weights with single-cell pathway activities, providing a powerful framework for deciphering microenvironment-mediated mechanisms in complex diseases.

Notwithstanding its strengths, scPagwas presents several limitations. First, its performance is contingent on the comprehensiveness of GWAS summary data and the precision of single-cell annotation. Second, its reliance on predefined pathway gene sets (e.g., KEGG) may overemphasize established biological knowledge (47), potentially obscuring novel regulatory circuits. Furthermore, genome-wide linkage disequilibrium (LD) analysis entails considerable computational overhead. In comparison, machine learning methods are adept at identifying data-driven patterns—such as non-coding RNA interactions—though they often lack mechanistic interpretability (48). Future research could investigate hybrid frameworks that merge the strengths of both approaches, improving the detection of novel biological modules while retaining mechanistic clarity.

Our multi-omics investigation identified considerable dysregulation and functional heterogeneity of UNC93B1 in pancreatic cancer, with expression levels significantly elevated in metastatic lesions relative to primary tumors. Integrated profiling suggests that UNC93B1 contributes to tumor progression through multifaceted mechanisms. On one hand, its correlation with cell cycle and metastatic traits implies non-canonical modulation of malignant behaviors. On the other, strong associations with the cGAS-STING pathway indicate a potential role in shaping the immune landscape via interference with innate immune signaling. Prior work has established UNC93B1 as a regulator of TLR7/9 trafficking in antiviral immunity (49, 50), while TLR5/7 activation has been connected to EMT induction (51). Structurally, UNC93B1 is a polytopic transmembrane protein with 12 membrane-spanning domains, suggesting potential roles as a molecular chaperone or ion channel capable of integrating diverse signals via conformational shifts. This raises the hypothesis that it may directly regulate EMT master transcription factors (e.g., SNAI1) through effects on nuclear transport or protein stability, thereby accelerating dissemination. Its overexpression in pancreatic cancer may be attributable to STING downregulation (25, 52)—paralleling known TLR regulatory mechanisms—leading to suppression of cGAS-STING pathway activity.

The cGAS-STING axis exhibits context-dependent roles in oncology, including induction of the senescence-associated secretory phenotype (SASP) and dual effects on tumor control (53). During senescence, elevated CDK inhibitors such as p16INK4A (34) induce cell cycle arrest while promoting morphological alterations and membrane integrity loss. As a cytosolic DNA sensor, cGAS-STING activation—triggered by genomic instability or mitochondrial damage—can provoke senescence or apoptosis. Paradoxically, although pathway activation can restrain proliferation via senescence, senescent cells frequently develop resistance to apoptosis (54), which may compromise therapeutic outcomes. Beyond senescence, cGAS-STING exerts dichotomous effects: hyperactivation may drive immunosuppression via aberrant IFN-α production, accelerating metastasis, whereas physiological stimulation typically induces IFN-β-dominated type I interferon responses that enhance NK and T-cell mediated antitumor immunity.

Immune evasion—a recognized cancer hallmark (22)—is exemplified by STING loss in colorectal (55), gastric (56), and melanoma (57) malignancies, which disrupts IFN-mediated tissue repair and T-cell recruitment, thereby promoting tumorigenesis. cGAS deficiency in colorectal cancer models attenuates type I interferon production, reducing infiltration of CD8+ T cells and NK cells, while STING knockout mice display compromised antitumor immunity and accelerated tumor growth (58).

Pseudotemporal trajectory analysis indicated progressive upregulation of UNC93B1 along with terminal differentiation phenotypes, suggesting its role as a “molecular timer” in cell fate determination. GSVA enrichment further linked UNC93B1-associated networks to RIG-I-like receptor (RLR) and Hedgehog (Hh) signaling. Although RLRs primarily sense viral RNA, Hh signaling—while not directly tied to UNC93B1—maintains pancreatic cancer stemness via SOX9 regulation (59). As a transmembrane transporter, UNC93B1 could potentially influence cancer stem cell traits by modulating SMO receptor localization or Gli transcription factor nuclear translocation, though this speculative model requires experimental confirmation beyond the current study.

Through integrated multi-omics analysis, we established a correlation between elevated UNC93B1 expression and adverse prognosis in pancreatic cancer patients. Subsequent evaluation of UNC93B1 in pancreatic cancer cell lines revealed relatively high expression in Capan-1 cells, which harbor the KRAS G12D mutation—one of the most frequent KRAS variants in this malignancy. Notably, Capan-1 exhibits higher metastatic propensity compared to BxPC-3 and Panc-1 lines (60). The elevated UNC93B1 expression in Capan-1 implies a pro-tumorigenic function. Indeed, UNC93B1 knockdown markedly impaired migration, invasion, proliferation, and clonogenicity, indicating its role as a key driver of pancreatic cancer progression. In the EMT process, CDH1 (E-cadherin) and VIM (vimentin) serve as central regulators. CDH1 mediates calcium-dependent cell-cell adhesion, while VIM maintains mesenchymal cytoskeletal integrity. During EMT, CDH1 downregulation weakens intercellular adhesion and facilitates dissemination, whereas VIM upregulation enhances motility and invasiveness. Pathway analysis indicated that UNC93B1 knockdown elevated CDH1 and suppressed VIM, thereby attenuating EMT. However, the precise mechanism underlying UNC93B1-mediated EMT regulation in Capan-1 remains unclear. Although current evidence does not support direct cGAS-STING regulation of EMT, prior studies suggest potential crosstalk between TLR signaling and Wnt or endoplasmic reticulum stress pathways—possibly modulated by UNC93B1’s transmembrane topology—as plausible EMT regulatory routes (61–63). Thus, UNC93B1 may modulate EMT in Capan-1 either autonomously or through TLR downstream effectors, though detailed mechanisms warrant further experimental dissection.

In this study, knockdown of UNC93B1 markedly suppressed PDAC cell proliferation, migration, and clonogenic capacity in immune cell–free *in vitro* models, indicating that the cGAS–STING pathway may exert a cell-autonomous, immune-independent tumor-suppressive function in pancreatic cancer. This finding is consistent with the growing recognition of non-immunological roles of cGAS–STING signaling. Beyond its canonical function in activating innate immunity, accumulating evidence suggests that this pathway can directly restrain cell growth and malignant phenotypes within tumor cells. Mechanistically, sustained activation of cGAS–STING signaling can induce cellular senescence. In our study, UNC93B1 knockdown resulted in a significant upregulation of the senescence marker p16^INK4a^, accompanied by G1-phase cell cycle arrest, suggesting that this pathway may mediate stable cell cycle inhibition through the p16^INK4a^/Rb or p53/p21 axis, in line with previous reports (64, 65). In addition, UNC93B1 depletion was associated with features of EMT reversal, including upregulation of the epithelial marker CDH1 and downregulation of the mesenchymal marker VIM, indicating that cGAS–STING signaling may directly limit tumor cell migratory capacity by suppressing EMT-related transcriptional programs. Taken together, the pro-tumorigenic role of UNC93B1 in PDAC likely involves both modulation of the tumor microenvironment and tumor cell–intrinsic mechanisms. The *in vitro* and immunodeficient *in vivo* data presented here primarily highlight its critical function in promoting malignant phenotypes through suppression of cGAS–STING–mediated intrinsic restraints on cell proliferation and migration. These findings provide a new perspective on the role of the cGAS–STING pathway in pancreatic cancer, an archetypal “immune desert” tumor (66).

Concurrently, we observed that UNC93B1 knockdown activated the cGAS-STING pathway, augmenting transcription of downstream mediators (IFN-β and CXCL10) and promoting cellular senescence. Cell cycle analysis by flow cytometry revealed G1 arrest in sh-UNC93B1 cells, potentially due to p16INK4a induction following cGAS-STING activation, inhibiting CDK4/6 activity. In xenograft models, UNC93B1 depletion suppressed tumor growth and tumorigenicity. Immunohistochemistry confirmed cGAS-STING pathway activation in UNC93B1-knockdown tumors, accompanied by elevated IFN-β and CXCL10 within the tumor microenvironment. Although nude mice lack T cell-mediated adaptive immunity, prior studies indicate that CXCL10 can recruit NK cells (67–69). We thus speculate that UNC93B1 knockdown may augment NK cell-dependent immunosurveillance via inflammatory cytokine induction, partly accounting for the observed tumor suppression. This hypothesis, however, requires validation through NK cell depletion or co-culture assays.

In summary, the rapid and aberrant proliferation characteristic of tumors can induce genomic instability—including chromosomal aberrations, deletions, and breaks (70–72)—which may activate the cGAS-STING pathway (73) and provoke senescence-like phenotypes and inflammatory cytokine secretion to restrain tumor growth. Our findings indicate that highly expressed UNC93B1 inhibits STING protein, attenuating cGAS-STING pathway activity, thereby potentially enhancing pancreatic cancer aggressiveness. Hence, we propose that UNC93B1 likely plays a pivotal role in disease progression.

This study employed multi-omics integrative strategies to elucidate the critical function of UNC93B1 in pancreatic cancer, yet several limitations should be addressed in future work. The scPagwas algorithm depends on the completeness of GWAS summary statistics and accuracy of single-cell annotations. The GWAS dataset (bbj-a-140) used here primarily comprises East Asian individuals, which may introduce population bias and limit generalizability to other ethnic groups. Moreover, single-cell data were generated across heterogeneous sequencing platforms (HiSeq 4000 vs. NovaSeq 6000). Although Harmony was used for batch correction, residual technical noise may persist due to inter-platform differences in sensitivity and gene coverage. While algorithms such as LASSO and random forests enable efficient feature gene selection, their “black-box” characteristics hinder elucidation of gene regulatory networks. GSVA analysis relies on prior pathway databases (e.g., KEGG), potentially overlooking pancreatic cancer-specific regulatory circuits such as lncRNA-mediated signaling axes. This study focused predominantly on the Capan-1 cell line (KRAS G12D mutant), yet pancreatic ductal adenocarcinoma exhibits substantial genomic heterogeneity (e.g., diverse KRAS mutation subtypes, varying TP53 status) and microenvironmental complexity. A single cell line may not fully capture the functional diversity of UNC93B1 in PDAC, necessitating validation in patient-derived primary cells or organoid models. Finally, although UNC93B1 knockdown inhibited EMT and activated the cGAS-STING pathway, a direct causal link between EMT and cGAS-STING activation remains unverified and merits further experimental investigation. While multi-omics integration has revealed a critical role for UNC93B1 in pancreatic cancer, its biological mechanisms and clinical relevance warrant continued interdisciplinary exploration.

## 5 Conclusion

In summary, UNC93B1 is significantly upregulated in pancreatic tumor tissues relative to non-neoplastic counterparts, and its elevated expression is correlated with unfavorable patient outcomes. Functionally, UNC93B1 drives malignant progression by enhancing tumor cell proliferation, reinforcing invasive potential, and mitigating cell cycle arrest. Mechanistic investigations suggest that UNC93B1 likely contributes to an immune-tolerant microenvironment and counteracts senescence in pancreatic cancer cells, primarily through suppression of the cGAS-STING signaling pathway. Furthermore, UNC93B1 appears to mediate crosstalk between neoplastic cells and their microenvironment, as evidenced by the finding that its knockdown effectively inhibits the epithelial-mesenchymal transition (EMT) process.

## Data Availability

The original contributions presented in the study are included in the article/**Supplementary Material**. Further inquiries can be directed to the corresponding authors.

## Supplementary material

Supplementary Figure 1 Spatial expression pattern of UNC93B1 in pancreatic cancer tissues based on spatial transcriptomic data.
