# Supplementary figures and images for: UNC93B1 promotes pancreatic cancer progression through modulation of cGAS–STING signaling
Source: Front Immunol. 2026 Feb 4;17:1718849. doi: 10.3389/fimmu.2026.1718849 (PMC12913547; doi:10.3389/fimmu.2026.1718849)

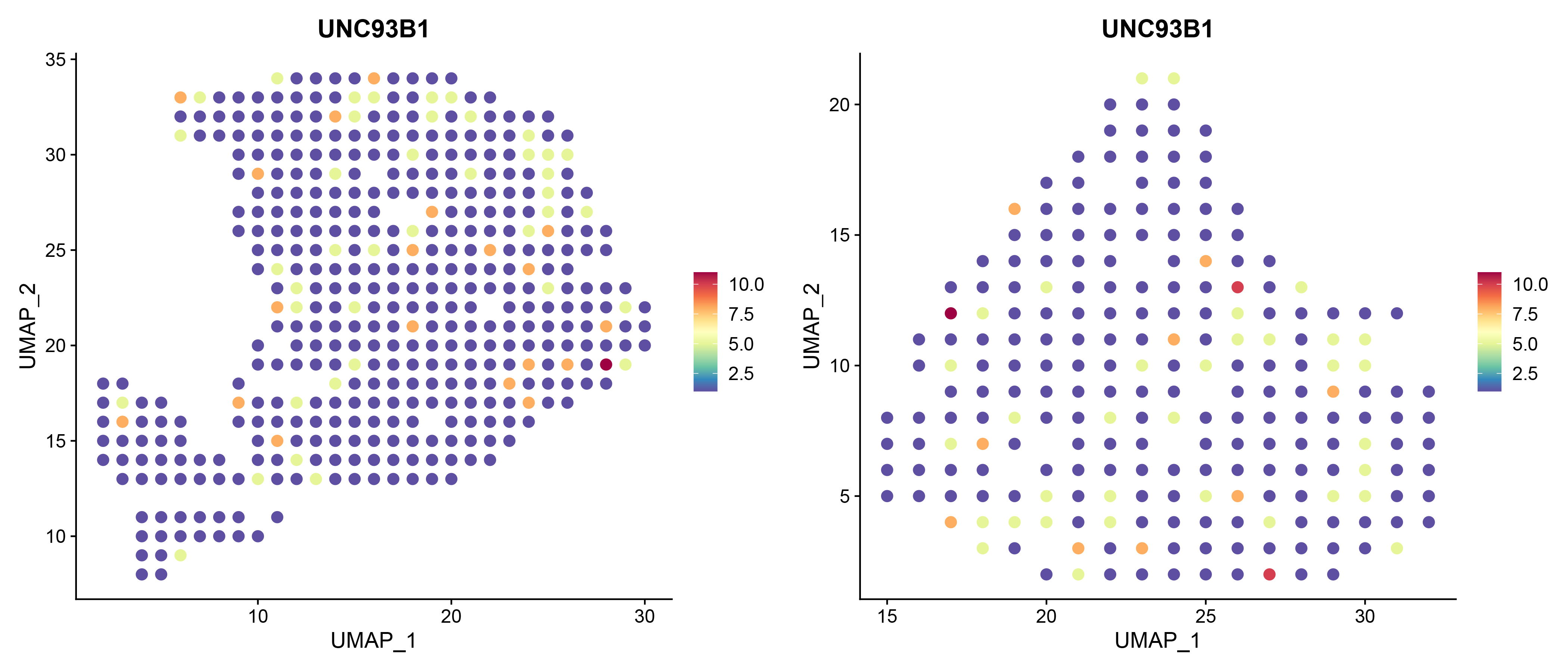

Supplement: Supplementary Figure 1 — Spatial expression pattern of UNC93B1 in pancreatic cancer tissues based on spatial transcriptomic data. [file Image1.jpeg]
